# Climate warming reduces soil gaseous nitrogen losses in a temperate forest

**DOI:** 10.1073/pnas.2513401122

**Published:** 2025-11-24

**Authors:** Kai Huang, Di Wu, Dongwei Liu, Yihang Duan, Peter Dörsch, Klaus Butterbach-Bahl, Xiaoming Fang, Yuqi Liu, Chao Wang, Haoming Yu, Lingrui Qu, Jingwen Xu, Geshere Abdisa Gurmesa, Ronghua Kang, Shushi Peng, Erik A. Hobbie, Xiaotang Ju, Shuijin Hu, Oliver L. Phillips, Per Gundersen, Weixing Zhu, Peter M. Homyak, Yunting Fang

**Affiliations:** ^a^Chinese Academy of Sciences Key Laboratory of Forest Ecology and Silviculture, Institute of Applied Ecology, Chinese Academy of Sciences, Shenyang 110016, China; ^b^Department of Environmental Sciences, University of California, Riverside, CA 92521; ^c^Key Laboratory of Stable Isotope Techniques and Applications, Shenyang 110016, China; ^d^Qingyuan Forest Chinese Ecosystem Research Network, National Observation and Research Station, Liaoning Province, Shenyang 110016, China; ^e^Institute for Global Change Biology and School for Environment and Sustainability, University of Michigan, Ann Arbor, MI 48109; ^f^Faculty of Environmental Sciences and Natural Resource Management, Norwegian University of Life Sciences, Ås 1432, Norway; ^g^Pioneer Center Land-Center for Landscape Research in Sustainable Agricultural Futures, Agroecology, Aarhus University, Aarhus C 8000, Denmark; ^h^University of Chinese Academy of Sciences, Beijing 100049, China; ^i^Laboratory for Earth Surface Processes, College of Urban and Environmental Sciences, Institute of Carbon Neutrality, Peking University, Beijing 100871, China; ^j^Sino-French Institute for Earth System Science, College of Urban and Environmental Sciences, and Laboratory for Earth Surface Processes, Peking University, Beijing 100091, China; ^k^Earth Systems Research Center, Morse Hall, University of New Hampshire, Durham, NH 03824; ^l^School of Tropical Agriculture and Forestry, Hainan University, Haikou 570228, China; ^m^Department of Entomology and Plant Pathology, North Carolina State University, Raleigh, NC 27695; ^n^School of Geography, University of Leeds, Leeds LS2 9JT, United Kingdom; ^o^Department of Geosciences and Natural Resource Management, University of Copenhagen, Frederiksberg C 1958, Denmark; ^p^Department of Biological Sciences, Binghamton University, State University of New York, Binghamton, NY 13902

**Keywords:** experimental warming, nitric oxide, nitrous oxide, N cycling, temperate forest

## Abstract

Global warming is projected to accelerate ecosystem nitrogen (N) loss via gaseous pathways, thereby decreasing N availability, a critical nutrient for primary productivity and carbon sequestration. However, the models forecasting this ecosystem N loss are based on laboratory experiments that are inherently uncertain and have had few in situ validations. Over 6 y, we measured ~200,000 soil nitric oxide (NO; an air pollutant) and nitrous oxide (N_2_O; a powerful greenhouse gas) fluxes and used an upscaling approach to estimate N_2_ fluxes after warming a temperate forest by 2 °C. Against thermodynamic theoretical predictions, warming unequivocally lowered emissions of NO by 19% and of N_2_O by 16%. These lower gaseous N losses were not explained by complete reduction of NO and N_2_O to N_2_, leaching, nor plant uptake, but rather by the warming-induced drying of soils that constrained microbial activity, consistent with other warming experiments where precipitation was less than 1,000 mm y^−1^. Our findings challenge ecosystem model assumptions where warming, alone, accelerates N emissions and underscores how warming-induced losses in soil moisture and shortened freeze-thaw periods can offset temperature effects. Our data underscore the need for explicit consideration of in situ soil moisture when predicting changes on the terrestrial N cycle as the planet warms.

## Significance

Climate models project that under future warming scenarios, ecosystems will lose nitrogen (N) because of accelerated cycling and increased gaseous N losses. However, these projections are predominantly derived from laboratory experiments that may not accurately represent field conditions. Opposite to these projections, our 6-y field warming experiment revealed soil N emissions were suppressed under warming, an observation shared across other warming experiments receiving less than 1,000 mm of yearly precipitation. This reduction in soil N emissions was mechanistically linked to lower microbial processing of N as soil moisture decreased under warming. Our results underscore how warming-induced losses in soil moisture can offset expected temperature effects on soil N cycling as the planet warms.

Nitrogen (N) is an essential nutrient often limiting plant primary productivity and the capacity of forests to sequester carbon (C), yet mounting global evidence suggests N availability across terrestrial ecosystems is declining (1, 2). One reason for the decline may include the accelerated emissions of nitric oxide (NO; an air pollutant at high concentrations in the troposphere and indirect greenhouse gas), nitrous oxide (N_2_O; a powerful greenhouse gas and major driver of stratospheric ozone destruction), and dinitrogen gas (N_2_; inert gas and major atmospheric constituent) from soils as global temperatures rise (3–5), which summed together can account for half of the N inputs to forest ecosystems (6, 7). Among these gases, the accelerated emission of N_2_O from soil is particularly concerning due to its potential to stimulate further warming and, hence, increased soil emissions (i.e., development of a positive feedback) (3–5). Models used to predict how soil N emissions may change in response to warming are often built on thermodynamic theory (i.e., the Arrhenius equation) (8–12), in which N emission rates increase exponentially with temperature as observed in laboratory studies (12, 13). However, experimental field studies available to validate these assumptions are rare, as developing and maintaining field warming sites is resource-intensive and making in situ N gas measurements present many challenges (14–18). Among the major gaseous N species (NO, N_2_O, and N_2_), N_2_O has been studied the most, presumably because of its role as a greenhouse gas and driver of stratospheric ozone destruction. Nevertheless, only ten forest sites worldwide have been monitored to examine how in situ warming affects N_2_O emissions, producing a wide range of responses (*SI Appendix,* Fig. S1). In contrast to N_2_O, field studies assessing the impact of warming on NO emissions from forest soils do not exist, and there are no in situ observations on how warming affects N_2_ emissions, as direct field measurements of N_2_ flux are still not feasible due to the large N_2_ atmospheric background.

Increasing global temperatures are expected to alter soil N cycling both directly and indirectly. Directly, warming affects microbial metabolism via enzymatic kinetics; enzyme activity increases with temperature (10, 12). Indirectly, warming can lower soil moisture by increasing evapotranspiration rates (19, 20), which decreases substrate solubility and diffusion and, thus, microbial access to N (21, 22). As soil moisture is a key factor governing microbial community structure, enzyme activities, and soil oxygen availability (23, 24), even small changes in soil moisture may dramatically alter the response of gaseous N production and consumption to warming (25, 26). However, interactions between changing temperature and soil moisture on the N cycle under climate warming are not well constrained (27, 28). Disentangling how temperature and moisture interact to control gaseous N emissions in situ as temperatures increase is necessary to predict N availability in forest ecosystems, forecast future emissions of NO and N_2_O, and predict the capacity of forests to sequester C.

**Fig. 1.**
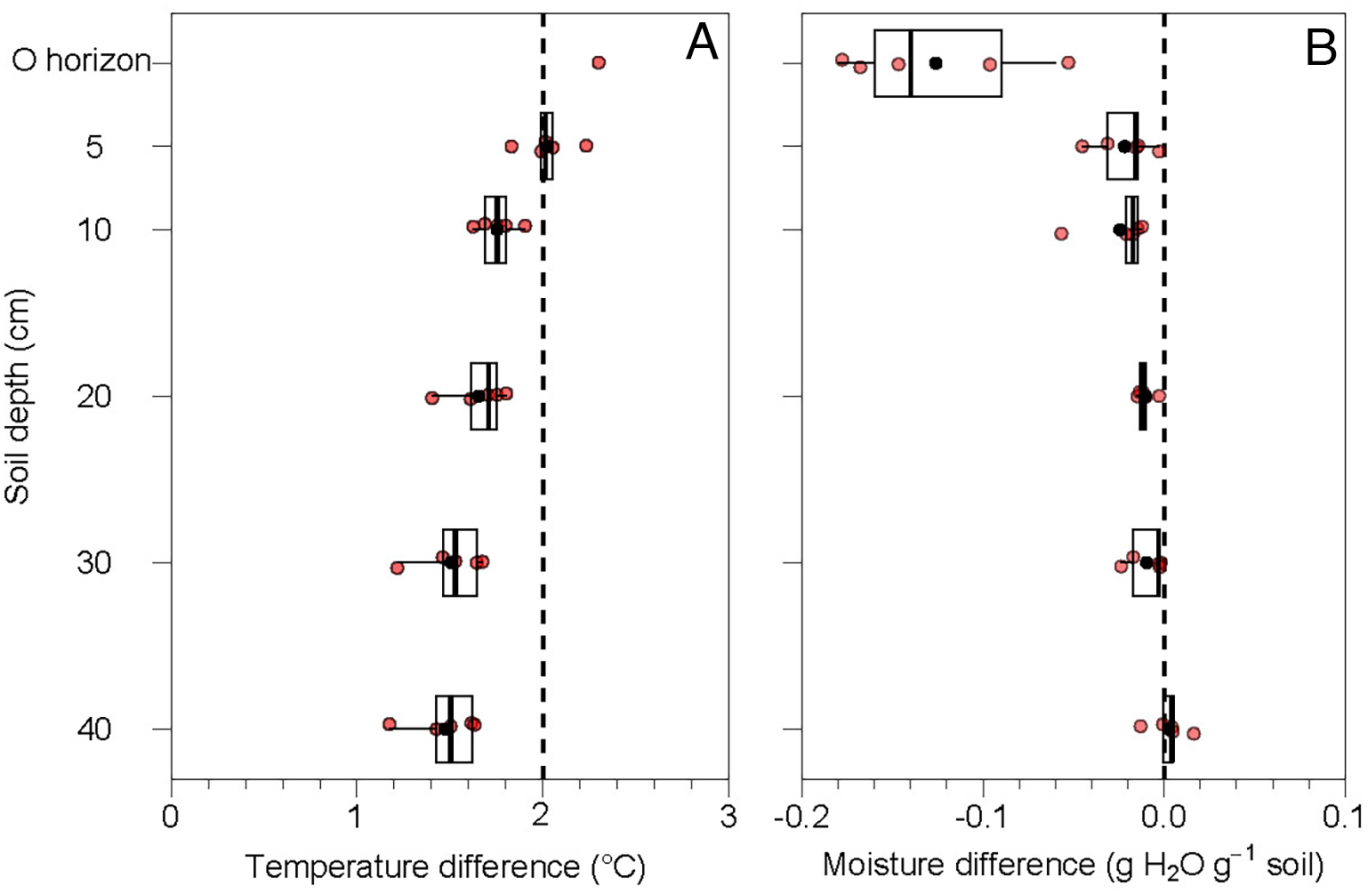
Warming effects on (*A*) soil temperature and (*B*) moisture as a function of depth. Observations were made from May to October in 2019-2023 when heaters were active. Soil moisture in the O horizon was measured during sampling, complemented by automated temperature monitoring from 2023 onward. Red jittered dots represent annual means for each soil depth, black dots represent the average values at each depth (n = 5 y). Box plots are standard Tukey plots, where the center line represents the median, the lower and upper lines represent the first and third quartiles, and whiskers represent +1.5 times the interquartile range.

To this end, we conducted a 6-y forest soil warming experiment using arrayed infrared heaters (*SI Appendix,* Fig. S2). The heaters increased temperature by 2.0 ± 0.1 °C (hereafter, mean ± SE) in mineral soils measured 5 cm below the surface, and by 1.8 ± 0.1 °C to 1.5 ± 0.1 °C measured at 10 to 40 cm depths (Fig. 1*A*). In response to warming, soil moisture decreased in the organic surface horizon (O horizon) by 0.13 ± 0.02 g H_2_O g^−1^ soil and by 0.02 ± 0.01 g H_2_O g^−1^ soil in mineral soils 0 to 10 cm below the surface, representing soil moisture reductions of 16% and 5% compared to ambient (Fig. 1*B*). We performed over 200,000 in situ measurements of soil NO and N_2_O fluxes and approximated N_2_ fluxes using an in situ ^15^N tracing experiment (29) that was scaled up based on the ratios of N_2_:N_2_O and N_2_O flux measurements at our site (*SI Appendix,* Fig. S3). We also quantified soil net N mineralization and nitrification rates and analyzed several chemical and biological factors known to influence soil N transformations.

**Fig. 2.**
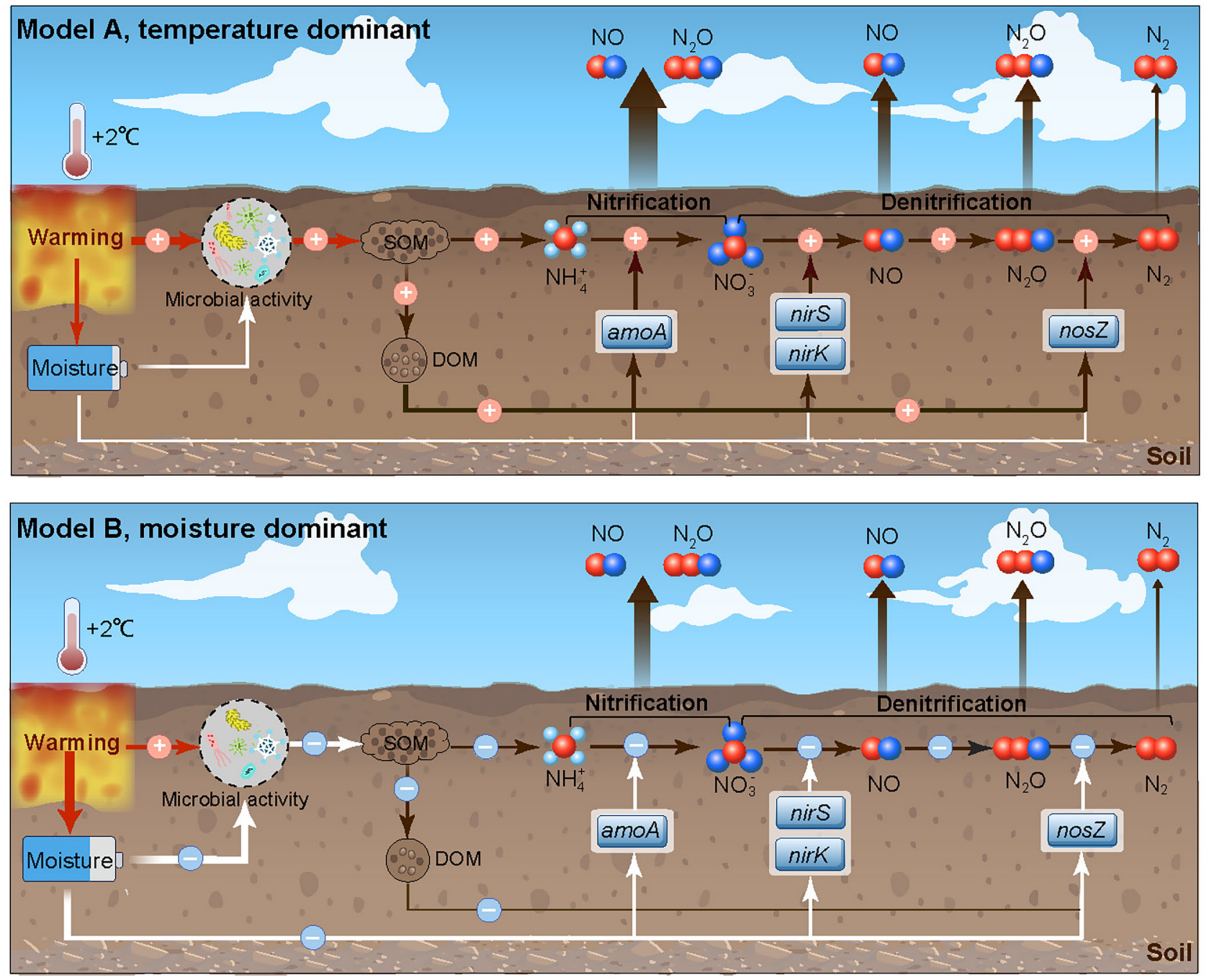
Conceptual models illustrating the response of gaseous N emissions in forest soil to warming. (*A*) warming favors SOM decomposition, increasing dissolved organic matter (DOM) and soil N transformations through changes in microbial activity. (*B*) warming reduces soil moisture, subsequently reducing SOM decomposition and N transformations. The thickness of the arrows represents the relative size of the effect. A positive effect is denoted by “+” and a negative effect by “−”.

We hypothesized that warming would increase soil N emissions as predicted by thermodynamic theory (Model A, temperature-response dominant, Fig. 2). Alternatively, we hypothesized that warming-induced losses in soil moisture would offset or even override the temperature effect and lower N emissions (Model B, Moisture-response dominant, Fig. 2). We found that warming lowered NO emissions by 19% and N_2_O emissions by 16%, supporting the alternative hypothesis. These lower N emissions were associated with the drying of surface soils under warming that constrained soil N mineralization and nitrification rates. Our measurements show that the warming-induced drying of surface soils can override the temperature sensitivity of gaseous N emissions as predicted from thermodynamic theory and laboratory experiments, suggesting in situ soil moisture is a critical factor for models to simulate and predict changes in soil N emissions as the planet warms.

## Results

### Warming Effects on Soil N Emissions.

**Fig. 3.**
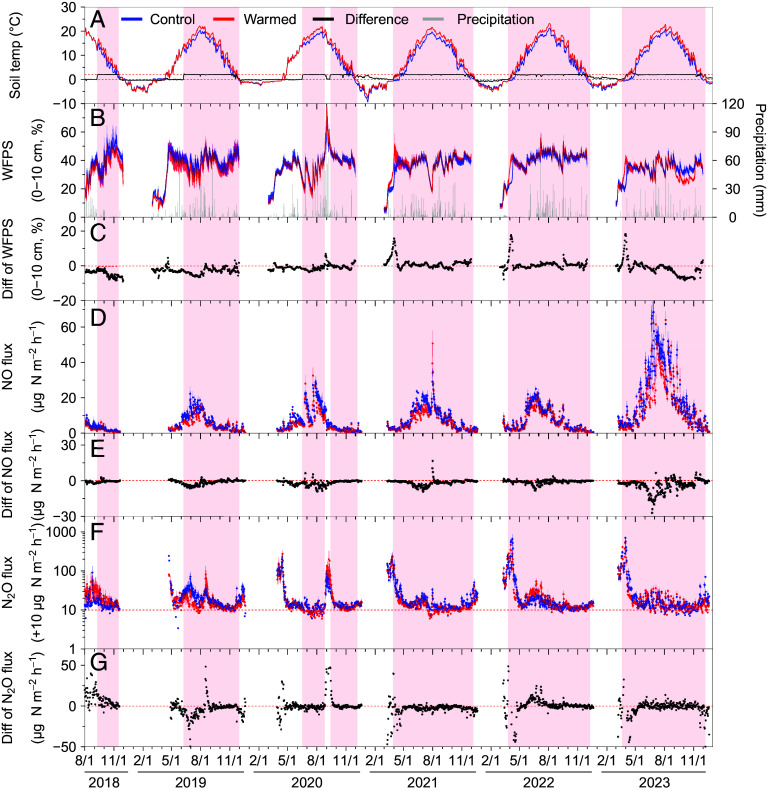
Dynamics of soil NO and N_2_O fluxes from control and warmed soils over 6 y. Soil temperature in mineral soils at 5 cm depth (*A*), soil water-filled pore space (WFPS) in mineral soils (0 to 10 cm depth) and daily precipitation (*B*), the difference in WFPS between warmed and control treatments (*C*), NO fluxes (*D*), differences in NO flux between warmed and control treatments (*E*), N_2_O fluxes (*F*), and differences in N_2_O flux between warmed and control treatments (*G*). Values for warmed plots are presented in red and from control plots in blue. In panel (*A*), the black solid line denotes the temperature difference between warmed and control treatments (i.e., the warming effect). The light red background represents the warming period in all the panels. The values are the daily means of three warmed and control plots, and all the values are mean ± SE (n = 3 plots).

**Fig. 4.**
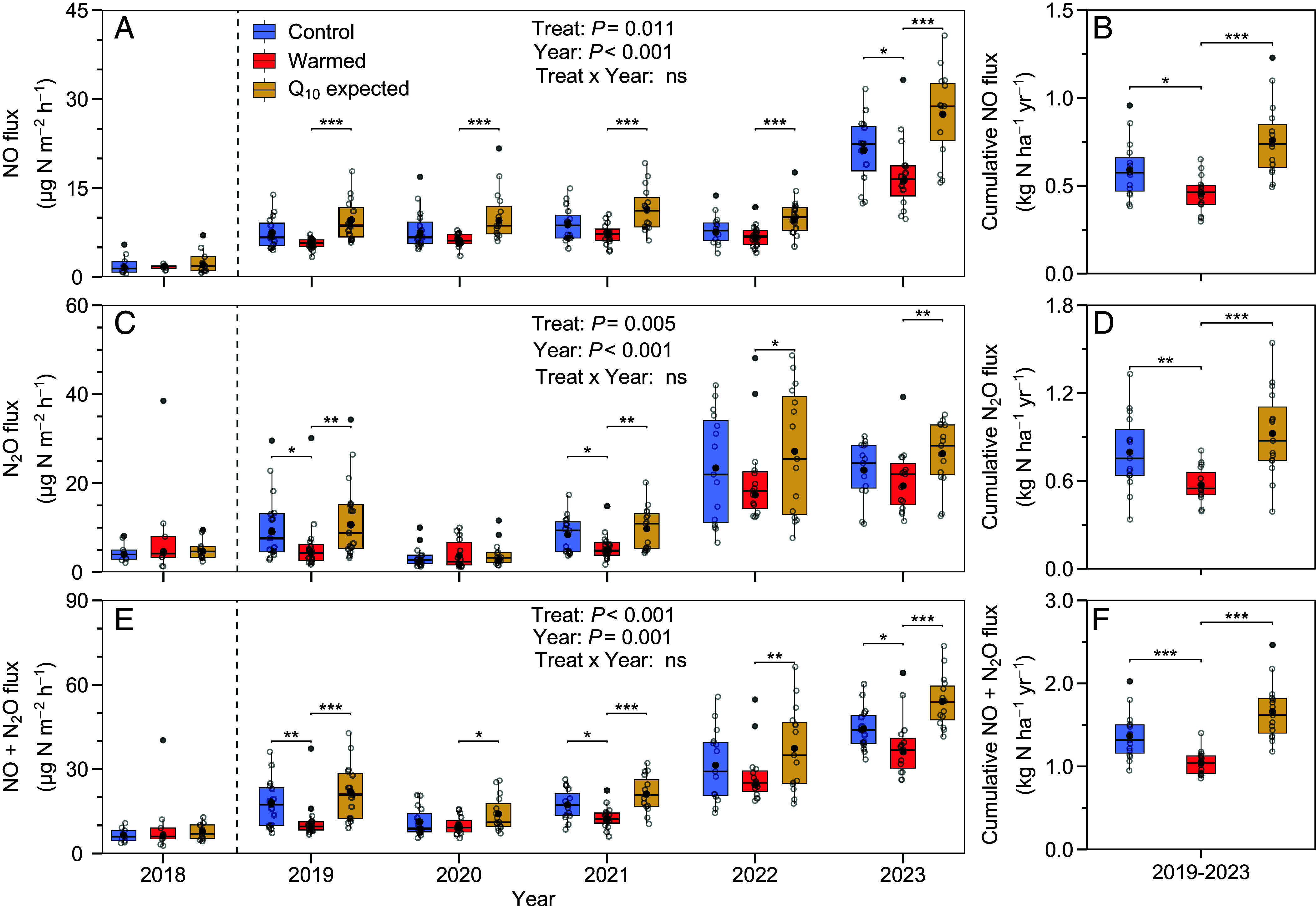
Soil NO and N_2_O fluxes during experimental warming. Warming periods extended from March (2021-2023) or June (2019-2020) to December. Average NO fluxes from 2018 to 2023 (*A*), cumulative NO fluxes from 2019 to 2023 (*B*), average N_2_O fluxes (*C*), cumulative N_2_O fluxes (*D*), average NO+N_2_O fluxes (*E*), and cumulative NO+N_2_O fluxes (*F*). Q_10_-expected fluxes were calculated based on their Q_10_ values. Colors represent different treatments (blue = control, red = warming, and orange = Q_10_-expected-fluxes). Gray dots represent the average values for each chamber, while black dots represent the average values for each group (n = 15 chambers). Repeated measures ANOVA was used to test the effects of warming treatment and time on soil NO and N_2_O fluxes from 2019 to 2023. The statistical significance between control and warming treatment is indicated by asterisks (*P < 0.05, **P < 0.01, and ***P < 0.001) or ns (nonsignificant).

Warming did not affect NO emissions during the first year of treatment in 2018. However, from 2019 to 2023, warming lowered the annual NO flux by 19 ± 2% (mean ± SE) relative to the control to 0.6 ± 0.2 kg N ha^−1^ y^−1^ (*P* = 0.01, Figs. 3 and 4 *A* and *B*). Occasionally, warming increased NO emissions above the control shortly after heavy precipitation events (≥30 mm) (*SI Appendix,* Fig. S4).

**Fig. 5.**
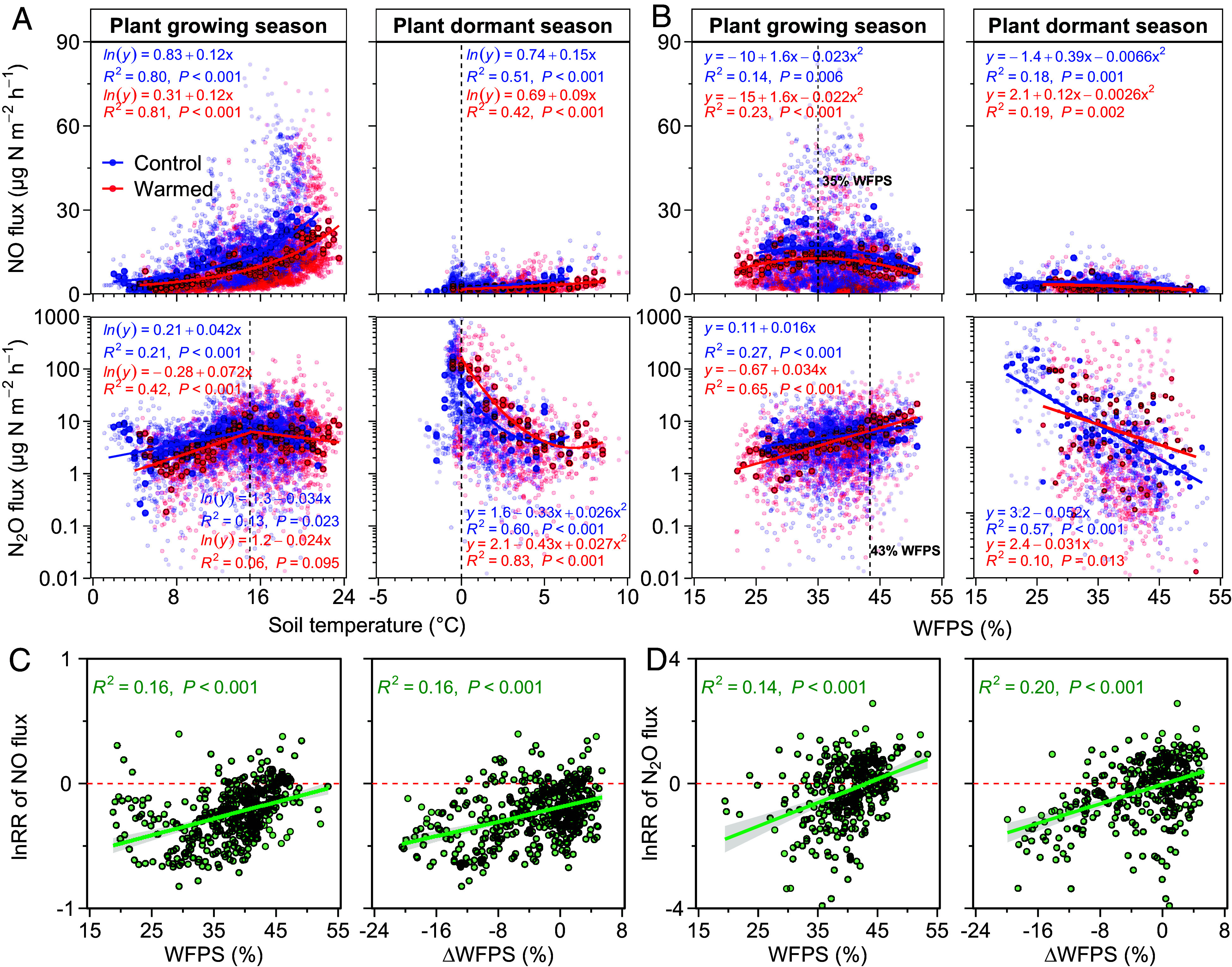
The relationship of NO and N_2_O fluxes with soil temperature and moisture (WFPS) in mineral soils (0 to 10 cm depth). Data from periods when the warming experiments were inactive, spanning from 2019 to 2023, are not included. Seasons were divided into plant growing season (May to October) and plant dormant season (November to April). Red and blue lines represent fitting relationships between NO or N_2_O fluxes and soil temperature or moisture for the warmed and control groups. The fitting relationships were analyzed using the recalculated data (bold dots) binned at intervals of 0.5 °C for temperature or 1% WFPS for moisture (*A* and *B*). The relationship of warming response ratio (lnRR) of NO and N_2_O fluxes with initial WFPS and the change in soil moisture (ΔWFPS) induced by warming in mineral soils (0 to 10 cm depth) was examined from June to August (*C* and *D*).

Warming also lowered the annual N_2_O flux during 2019-2023 by 16 ± 8% relative to the control to 0.8 ± 0.2 kg N ha^−1^ y^−1^ (*P* = 0.01, Fig. 4 *C* and *D*). N_2_O emissions from warmed plots were also sometimes greater than the control when soil moisture was high, both in the early spring when soils thawed and after heavy precipitation (Fig. 3 and *SI Appendix,* Fig. S4). This produced a significant positive correlation between the response of N_2_O emissions to warming and the change in soil water-filled pore space (ΔWFPS; a metric representing the change in soil moisture in response to warming) (Fig. 5*D*).

The warming effect on N_2_ emissions was estimated using an in situ scaling approach (*Materials and Methods*). During the plant growing season (May to October), soil N_2_ emissions from control plots averaged 0.9 ± 0.1 kg N ha^−1^ y^−1^, and did not change significantly in response to warming (*SI Appendix,* Figs. S3*C*, S5 *A* and *B*, and S6).

### Warming Effects on Soil N Cycling and Functional Genes.

**Fig. 6.**
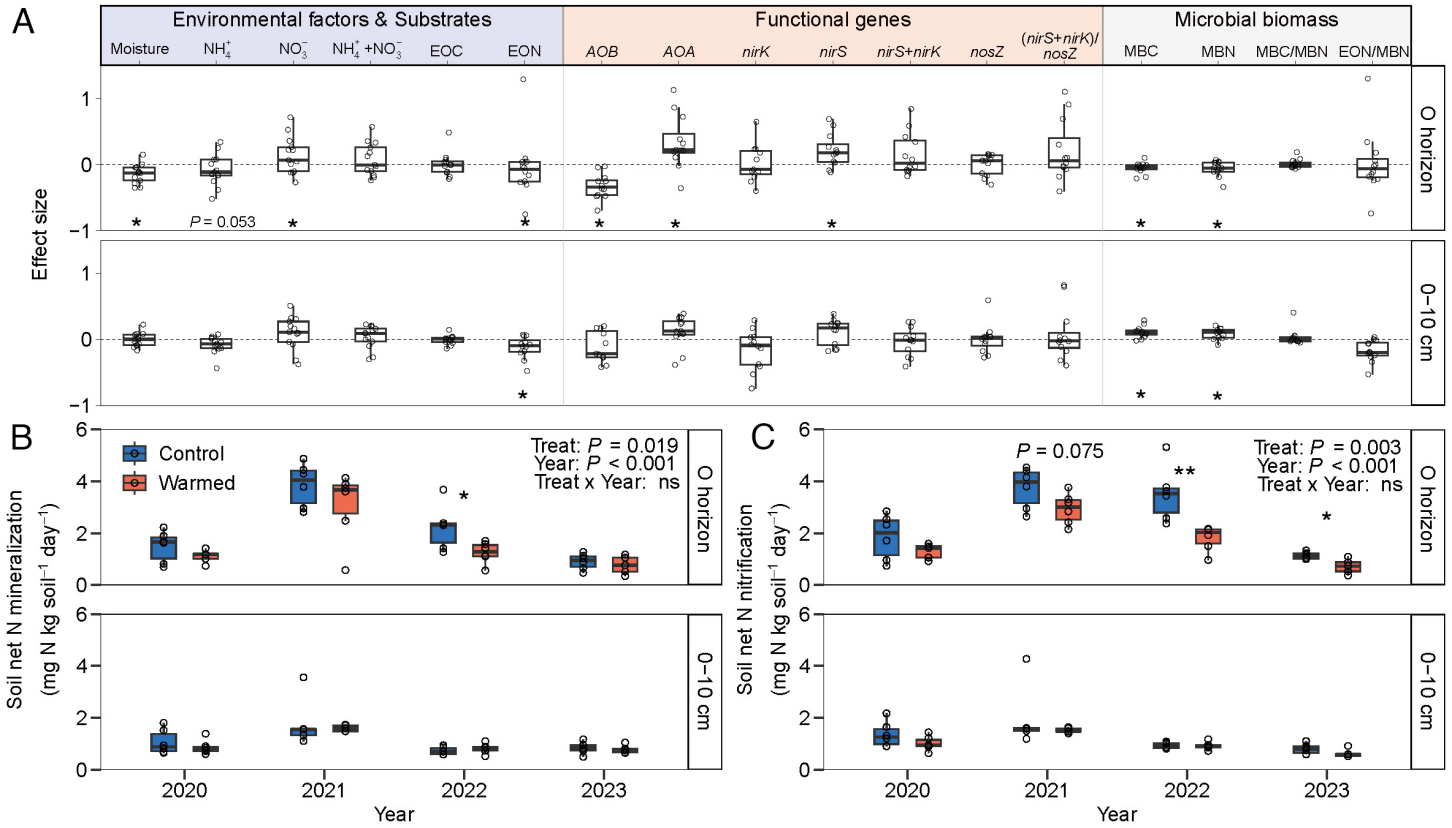
Response of soil N substrates, functional gene abundances, microbial biomass C and N, and net N transformation rates to warming. The effect sizes in response to warming on soil substrates, the functional genes, MBC, and MBN involved in nitrification and denitrification processes across all sampling times from 2019 to 2023 are shown (*A*). The dots represent net N mineralization and nitrification rates in the organic horizon and mineral soils (0 to 10 cm depth) during the plant growing season (May to October), and boxplots represent the confidence range of the observations (n = 6 subplots) (*B* and *C*). Repeated measures ANOVA was used to test the effects of warming treatment and time on soil net N mineralization and nitrification rates, and the relevant soil parameters. The level of significance is determined by the two-sided *t* test: *P < 0.05, **P < 0.01, and ***P < 0.001.

Soil extractable ammonium (NH_4_^+^) and nitrate (NO_3_^−^) concentrations varied seasonally and distinctly in response to 6 y of experimental warming (Fig. 6*A* and *SI Appendix,* Fig. S7). During the plant growing season, warming decreased NH_4_^+^ by 8 ± 7% in the O horizon, though this effect was only significant at *P* = 0.053. Relative to NH_4_^+^, the warming effect on NO_3_^−^ was more complex. Over much of the plant growing season (June to August), warming had no effect on NO_3_^−^ in the O horizon (*P* = 0.30), but at the onset of plant senescence (September to October), warming induced a positive effect (*P* = 0.02; *SI Appendix,* Fig. S7*C*). In contrast to the O horizon, warming did not significantly affect NO_3_^−^ nor NH_4_^+^ in the 0 to 10 cm mineral layer (*SI Appendix,* Fig. S7 *B* and *C*).

We further incubated soils in the laboratory across a temperature gradient to determine whether field warming changed soil NH_4_^+^ and NO_3_^−^ net production rates (i.e., net N mineralization and nitrification). Warming reduced soil net N mineralization by 21 ± 4% (*P* = 0.02) and net nitrification by 14 ± 6% (*P* = 0.01) in the O horizon during the plant growing season, but had no effect in the 0 to 10 cm mineral layer (Fig. 6 *B* and *C* and *SI Appendix,* Table S1). We also found that warming reduced both microbial biomass nitrogen (MBN) by 6 ± 3% (*P* = 0.02) in the O horizon and soil extractable organic N (EON) by 12 ± 5% (*P* = 0.01) in the 0 to 10 cm mineral layer (Fig. 6*A* and *SI Appendix,* Fig. S7 *E* and *G*).

Warming sometimes affected the abundance of functional genes associated with nitrification and/or denitrification like *amoA* encoding for NH_3_ monooxygenase in ammonia-oxidizing archaea (AOA) and ammonia-oxidizing bacteria (AOB), *nirS* and *nirK* encoding for NO_2_^-^ reductase, and *nosZ* encoding for N_2_O reductase (*SI Appendix,* Fig. S8). Specifically, across all sampling dates, warming increased the abundance of AOA *amoA* by 34 ± 12% (*P* < 0.001) and *nirS* by 22 ± 7% (*P* = 0.003), while decreasing the abundance of AOB *amoA* by 29 ± 6% in the O horizon (*P* = 0.04) (Fig. 6*A*). We did not detect a warming effect on the abundance of the N_2_O reductase functional gene (*nosZ*) (*SI Appendix,* Fig. S8*G*). As with net N mineralization and nitrification rates and extractable NO_3_^-^ and NH_4_^+^ in situ measurements, warming did not affect functional genes associated with either nitrification or denitrification in the 0-10 cm mineral layer.

### Ambient Soil NO and N_2_O Emissions (Control Plots).

Ambient soil NO fluxes ranged from 0.1 to 69 µg N m^−2^ h^−1^, with an average of 9 ± 0.3 µg N m^−2^ h^−1^ (n = 1,467 d) over the 6-y study period (Fig. 3*D*). Significant interannual variations were observed in NO emissions, which were ~2 to 3 times higher in 2023 compared to other years (Fig. 4*A*). Averaged across years, the annual NO emission was 0.6 ± 0.2 kg N ha^−1^ y^−1^, an estimate that assumes negligible fluxes during snow cover (i.e., December to March) (30, 31) (*SI Appendix,* Fig. S9 *A*–*D* and Table S2). NO emissions during the plant growing season (May to October) accounted for 85 ± 1% of the annual NO flux (*SI Appendix,* Fig. S9*D*).

Ambient soil NO emissions also varied seasonally, with higher emissions measured in the summer than in early spring and fall (Fig. 3*D*). This seasonal variation was related to ambient changes in soil temperature, which alone explained 80% of the seasonal variation during the plant growing season (Fig. 5*A*). Specifically, soil NO emissions increased exponentially with soil temperature, with an apparent temperature sensitivity (expressed by Q_10_) of 3.5 (*SI Appendix,* Fig. S10*A*). In addition to soil temperature, changes in ambient soil moisture also affected NO emissions during the plant growing season, producing some of the highest fluxes immediately after precipitation events (*SI Appendix,* Fig. S4 *B* and *C*), a common observation across ecosystems (21, 32). Ambient NO emissions were highest at 35% WFPS during the plant growing season, with emissions decreasing at both higher and lower WFPS according to a negative quadratic relationship established in the 0 to 10 cm mineral layer (Fig. 5*B*).

Ambient soil N_2_O fluxes were larger than NO fluxes, ranging from −7 to 697 µg N m^−2^ h^−1^, with a mean of 18 ± 1 µg N m^−2^ h^−1^ over the 6-y study period (Fig. 3*F*). Averaged across years, the annual N_2_O emission was 1.2 ± 0.2 kg N ha^−1^ y^−1^, roughly 105% higher than that for NO (*SI Appendix,* Table S2). In contrast to NO being highest in summer, the highest N_2_O emissions were often observed during freeze-thaw periods (March to April; *SI Appendix,* Fig. S9 *E*–*H*), with emissions peaking when soil temperatures were around 0 °C (Fig. 5*A* and *SI Appendix,* Fig. S4*D*). During plant dormant season (November to April), the average ambient soil N_2_O emission was 0.9 ± 0.3 kg N ha^−1^ y^−1^, which accounted for 78% of the annual emission (*SI Appendix,* Fig. S9 *E*–*H*). In line with previous studies (33, 34), N_2_O emissions during plant dormant season were negatively correlated with soil temperature and moisture (Fig. 5 *A* and *B*). In contrast, N_2_O emissions during the plant growing season were positively correlated with soil moisture and were maximized at 15 °C in the 0 to 10 cm mineral layer (Fig. 5 *A* and *B*).

## Discussion

We warmed soils in a temperate forest by 2 °C for 6 y and made over 200,000 in situ real-time NO and N_2_O emission measurements to understand how warming would interact with soil moisture to alter these emissions. Our data provide unequivocal evidence that experimental warming, without altering ambient precipitation, decreased soil NO and N_2_O emissions opposite to predictions from theoretical models (Fig. 4 and *SI Appendix,* Fig. S6). These in situ measurements challenge previous assumptions made from laboratory experiments that predict warming, alone, accelerates gaseous N loss (8–12). Here, we highlight the critical role of changing soil moisture under warming as a regulator of N emissions, particularly in ecosystems with mean annual precipitation of less than ~1,000 mm, underscoring the need for explicit consideration of in situ soil moisture when assessing the impacts of climate change on the terrestrial N cycle.

### Mechanisms for Reduced NO and N_2_O Emissions under Experimental Warming.

The lower soil NO and N_2_O emissions measured under warming contrasted expectations informed by soil laboratory experiments and theoretical models (Fig. 4). Based on Q_10_ functions developed from our observations in control plots, 2 °C of warming should have increased NO emissions by 28% and N_2_O by 16%, yet in situ NO and N_2_O emissions were instead 37% and 28% lower (Fig. 4 *B* and *D*). At least three possible mechanisms could explain the warming-induced decrease in NO and N_2_O emissions: 1) warming decreased the N_2_O/(N_2_O + N_2_) ratio by promoting the complete microbial reduction of NO and N_2_O to N_2_; 2) warming favored plant N uptake or N losses via leaching in response to increased mineralization of soil organic matter (SOM), lowering soil N availability; and/or 3) warming decreased soil moisture, suppressing microbial activity and, thus, the emission of N.

We ruled out that warming favored the complete reduction of NO and N_2_O to N_2_. Using an upscaling approach based on our in-situ ^15^N tracer experiment (see methods), we estimated 0.9 ± 0.2 kg N ha^−1^ y^−1^ were emitted as N_2_ from control plots (*SI Appendix,* Fig. S5 *A* and *B*), a number equivalent to the N_2_ emitted from the warmed plots (*P* = 0.40). The absence of a warming effect on N_2_ emissions is consistent with detecting no change in the abundance of the N_2_O reductase functional gene (*nosZ*) after warming our site (Fig. 6*A* and *SI Appendix,* Fig. S8*G*), as well as with a 16-y warming study in a European temperate forest, where the warming response of soil N_2_O fluxes diminished over time without promoting N_2_ emissions (15).

We also ruled out increased plant N uptake or N leaching lowered soil N availability and, thus, emission of both NO and N_2_O under warming. First, we found that warming did not increase the soil leaching volume, nor the N leached from the upper 40 cm of soil (NH_4_^+^: 0.2 ± 0.1 vs. 0.3 ± 0.1 mg N L^−1^ after warming; NO_3_^−^: 3.5 ± 0.4 vs. 3.0 ± 0.4 mg N L^−1^ after warming) (*SI Appendix,* Table S3). We then found that over the 6-y warming period, N did not increase in plant tissue, root biomass, woody biomass production, or litter yield (*SI Appendix,* Table S3), altogether suggesting that the third possibility, decreased soil moisture under warming, best explained the lower N emissions.

### Warming Dried Soils and Lowered N Emissions.

Our data suggest that the warming-induced 16% decline in surface soil moisture was enough to offset the expected temperature-dependent increases in NO and N_2_O emissions based on thermodynamic theory (35). Moisture-mediated suppression of N emissions was most pronounced for NO emissions, with decreases of 19% (growing season) and 27% (pregrowing season) under warming (*SI Appendix,* Fig. S9 *A*–*D*). To understand the mechanisms whereby lower soil moisture constrained NO emissions, we note that i) the molar ratio of NO:N_2_O emissions was greater than 1:1 in 60% of observations (an index indicative of nitrification controlling NO emissions (25, 36); *SI Appendix,* Fig. S3*B*) and that ii) there was a positive relationship both between NO flux and AOB *amoA* gene abundance and between the warming response of NO emission (lnRR) and NH_4_^+^ availability—i.e., the substrate for nitrification—in the 0-10 cm mineral layer (*SI Appendix,* Figs. S11 and S12). Altogether, these observations suggest NO emissions were primarily driven by nitrification, consistent with other studies (36, 37). We then found that net nitrification was positively associated with soil moisture in the O horizon and that as soil moisture decreased, so too did NO fluxes (*SI Appendix,* Fig. S11), a common observation across studies (38, 39). Indeed, the warming-induced lowering of soil moisture in the O horizon lowered net N mineralization by 21% and net nitrification by 14% in our laboratory incubations (*P* = 0.02 and *P* = 0.01; Fig. 6 *B* and *C*), linking a reduction in the substrate supply rates of mineral N under warming with lower NO and N_2_O emissions (22). Consistent with this understanding, when warming caused only small changes in soil moisture, as measured in the deeper 0 to 10 cm mineral layer (5% reduction in soil moisture), there was no effect on substrate supply rates required for NO production (i.e., net N mineralization and nitrification rates; Fig. 6*B*). Altogether, our data show warming-induced decreases in soil moisture constrained net N mineralization and nitrification rates, decreasing NH_4_^+^ availability and, thereby, suppressing NO production.

The response of N_2_O emissions to warming was also controlled by soil moisture and correlated with change in WFPS in the 0 to 10 cm mineral soil layer (ΔWFPS; i.e., the difference in WFPS between warmed and ambient soils) (Fig. 5 *B* and *D* and *SI Appendix,* Fig. S12). During the plant growing season, the negative response of N_2_O emissions to warming was consistent with the lower soil moisture and nitrification rates we measured, mirroring the substrate-controlled patterns observed for NO emissions (28). Soil warming reduced N_2_O emissions by 16% and 8% in both the plant pregrowing and growing seasons, with a more pronounced reduction (50%) observed in the plant postgrowing season (*SI Appendix*, Fig. S9 *E*–*G*). During spring freeze-thaw periods (March to April), warming advanced the timing of thawing by 8 to 11 d. Both soil moisture and N_2_O emissions increased immediately after thawing, but then N_2_O emissions decreased as soil moisture decreased (Fig. 3 and *SI Appendix,* Fig. S4). Thus, warmed soils thawed earlier and were less exposed to freeze–thaw cycles that are well known to stimulate denitrification-derived N_2_O emissions (40), helping to explain why N_2_O emissions decreased.

### Moisture Regulation of N_2_O Emissions across Experimental Forest Warming Studies.

To further explore how warming-induced changes in soil moisture affect in situ N_2_O emissions from forests—field experiments for NO do not exist—we summarized the ten studies that, to date, have evaluated this issue across Asia, Europe, and North America (*SI Appendix,* Fig. S1 and Table S4). Of these studies, the direction and magnitude of the N_2_O response (the standardized response ratio, RRn) to warming were governed by changes in soil moisture (*SI Appendix,* Fig. S13*L*), with a significant reduction in emissions when soil moisture decreased (R^2^ = 0.45; *P* = 0.001). We also found that, so far, warming-induced decreases in soil moisture mainly occurred in regions with mean annual precipitation of less than ~1,000 mm (*SI Appendix,* Fig. S13*O*), where warming-induced soil drying suppressed the expected increase in N_2_O emissions based on thermodynamic theory. Taken together, these observations further emphasize how approaches that exclusively consider accelerated microbial metabolism, while overlooking soil drying under warming, may not accurately capture soil N_2_O emissions.

### Ecological Implications.

Surface soil moisture during the plant growing season has decreased by 0.01 to 0.03 m^3^/m^3^ over the past four decades across the globe, particularly in temperate regions of Eurasia, representing reductions of 4 to 12% in average soil moisture (41, 42) that are expected to intensify (3, 43, 44). Recent studies show that the coupling between soil moisture and the atmosphere can favor future warming by reducing evapotranspiration and increasing solar shortwave radiation reaching the ground surface (45, 46). Despite the importance of this feedback, changes in soil moisture are often overlooked in forest warming studies. Our data underscore that changes in soil moisture and its effect on microbial dynamics regulate the response of gaseous N emissions to warming and must be explicitly incorporated into models.

As Earth warms, many studies expect accelerated N cycling and increased N emissions and, therefore, a positive warming feedback (12, 47, 48). At our site, we expected N emissions would increase by 20% using Q_10_-based estimates (*SI Appendix,* Fig. S5 *C* and *D*). However, increased N emissions in response to experimental warming have, so far, been constrained to regions where annual precipitation exceeds 1,000 mm to offset warming-induced losses in soil moisture (*SI Appendix,* Fig. S13). Consistent with our site receiving 811 mm annual precipitation, we show that warming reduced by 9% the average annual N flux (NO+N_2_O+N_2_) of 2.3 ± 0.4 kg N ha^−1^, implying that warming-induced decreases in soil moisture and shortened freeze-thaw periods, without a concomitant increase in precipitation, can override the expected temperature sensitivity of gaseous N emissions. Our results emphasize how changes in temperature, alone, may not reproduce the responses expected from laboratory-based studies, suggesting that overlooking changes in soil moisture under warming, may have significant implications for modeling forest soil N cycling responses to climate change, including future predictions of terrestrial carbon sink dynamics.

## Materials and Methods

### Study Site and Experimental Design.

The experimental warming site was located in the Qingyuan Forest CERN (National Observation and Research Station, Chinese Academy of Sciences), in Liaoning Province, Northeastern China (41°51′N, 124°54′E, 500 to 1,100 m elevation) (*SI Appendix,* Fig. S2*A*). The forest is a typical temperate mixed coniferous-broadleaf forest dominated by *Juglans mandshurica* and *Larix kaempferi* (49). The site is characterized by a continental monsoon climate, with a mean annual precipitation of 811 mm, more than 80% of which falls between May and September. Snowfall accounts for less than 6% of the annual precipitation. The mean annual temperature is 4.5 °C, and average daily extremes range from −37.6 to +36.5 °C, with an annual frost-free period of approximately 130 d (50). The plant growing season extends from May to late October, with snow typically covering soil from late November to late March. The annual atmospheric N deposition rate is 13.2 kg N ha^−1^ (51).

The warming experiment was designed in six 18 m × 6 m (108 m^2^) plots, three of which were randomly assigned to warming (*SI Appendix,* Fig. S2 *B* and *C*). The soil in the warming plots was irradiated by 18 infrared heaters (2 KW h^−1^, 8 mm diameter × 151 cm long, HS-2420 from Kalglo Electronics Co., Inc., USA) evenly spaced uniformly 2 m above the ground. In the ambient plots, identically shaped heaters were installed as sham devices. The estimated shading caused by these heaters is only about 3.8% of the total plot area, and the actual shading effect of the heaters would be much lower in a closed-canopy forest. During the snow-free period from late March to early December each year, we used an automatic temperature control system (Beijing Comity Measure & Control Co., Beijing, China) to achieve a constant warming effect of +2 °C above the control at a 5 cm depth in the mineral soil. No significant differences in aboveground biomass and soil properties were observed prior to initiation of the warming experiment (49).

### Field Observation of Soil NO and N_2_O Emissions.

We used an automated system to measure NO and N_2_O fluxes from August 2018 to December 2023. Each of the six plots contained 5 automated flux chambers (length × width × height = 0.4 m × 0.4 m × 0.5 m), made from transparent acrylic sheets. The chambers remained open except during flux measurements to allow the concentration of trace gases to return back to ambient conditions, ensuring spatially representative fluxes data (*SI Appendix,* Fig. S2*B*). A thermometer inside the center of each chamber recorded headspace temperature, while a small fan (6 cm × 6 cm × 2.5 cm) was used to maintain the air mixed. An automatic control system using an electric linear actuator (PRI-8600, Pri-Eco, Beijing, China) controlled the opening and closing of the chambers. Gaseous emissions were estimated from the change in concentration inside the chamber during 20-min chamber closures, during which chamber air was circulated through a nitrogen oxide analyzer (Model 42i-TL, Thermo Scientific, USA) and a greenhouse gas analyzer (Picarro-G2508, Picarro Inc., USA). The NO analyzer uses ozone to oxidize NO to NO_2_ prior to analysis and does not recirculate the vented gas from the analyzer back into the flux chamber. Fluxes from each chamber were measured every 5 h (4 to 5 times per day), and the daily emission rates of NO and N_2_O were calculated as an average of 72 fluxes from 15 chambers.

Soil NO and N_2_O fluxes were calculated as the sum of the concentration change over time (accumulation) and the difference between incoming and outgoing air (dynamic method) (52–54). These fluxes were calculated using the following Eq. **1**.

[1] $$\text{Flux}=\left[\frac{\text{dC}\cdot V}{\text{dt}\cdot A}+\;\frac{\left(C_{\text{out}}-C_{\text{in}}\right)\cdot Q}{A}\right]\cdot \frac{M}{V_{0}}\cdot \frac{P}{P_{0}}\cdot \frac{T_{0}}{T},$$

where $\frac{\mathrm{dC}}{\mathrm{dt}}$ is the rate of NO or N_2_O concentration (µg N m^−3^) change over time (h) from 6th to 10th min (5 min) for NO or 6th to 20th min (15 min) for N_2_O, during chamber closure, as estimated by linear regression; V is the total volume of the chamber, tubes, and analyzers (0.091 m^3^), A is the surface area covered by the chamber (0.18 m^2^), C_out_ and C_in_ are the average NO or N_2_O concentrations (µg N m^−3^) during the time interval of the regression, while the last 30 s after opening the chamber are used to record ambient NO or N_2_O concentrations (C_out_), Q is the air flow rate (3 L min^−1^) through the tubing, M is the molar mass of NO or N_2_O gases, T_0_ (273 K), V_0_ (22.4 L mol^−1^), and P_0_ (1,013 hPa) are standard temperature, volume, and pressure; T and P are the averaged air temperature (K) and pressure (hPa) inside the chamber. For the first 3 min of chamber operation, the lids were unlocked to empty the 20-m gas pipe. We used $\frac{\left(C_{\mathrm{out}}{-C}_{\mathrm{in}}\right)\cdot Q}{A}$ to calculate the dilution of the chamber headspace by ambient air, as ambient air was introduced into the chamber to replenish the gas discarded from the NO analyzer.

Six temperature probes (PT100, Comity Inc, Beijing, China) were installed at a 5 cm depth in mineral soils at each plot to minimize potential bias from spatial heterogeneity (*SI Appendix,* Fig. S2*C*), which provided the reference data for the temperature control system. Volumetric moisture content (0 to 10 cm mineral soil layer) of each plot was monitored using 10 uniformly placed probes (AP, Computer Network Information Center, Chinese Academy of Sciences, Beijing, China) based on frequency domain reflectometry (FDR) technology (55). Soil WFPS in the 0 to 10 cm soil mineral layer was calculated using volumetric water content (VWC), bulk density (BD, 0.70 g cm^−3^), and 2.65 as the specific density of quartz (g cm^−3^), according to Eq. **2**.

[2] $$\text{WFPS (\%)}\;=\frac{\text{VWC}}{1\;-\frac{\mathrm{BD}}{2.65}}\cdot 100.$$

To evaluate the effect of warming on soil moisture, changes in soil water-filled pore space (ΔWFPS) were calculated using WFPS values from both control and warming treatments, following Eq. **3**.

[3] $$\Delta \mathrm{WFPS}\;(\%)=\;\frac{{\mathrm{WFPS}}_{W}\;-\;{\mathrm{WFPS}}_{C}}{{\mathrm{WFPS}}_{C}}\cdot 100.$$

The apparent temperature sensitivity of NO or N_2_O fluxes was calculated as Q_10_, which denotes the relative increase in NO or N_2_O fluxes for a 10 °C increase in T. A few studies reported that the Q_10_ of NO emissions ranged from 2 to 4 over the temperature range of 0 to 35 °C, as measured in the laboratory (56). Previous incubation experiments with forest soils showed consistent Q_10_ values of 2.1 for N_2_O and 2.6 for N_2_ release by denitrification (12). At our study site, soil temperatures, based on continuous 6-y observations, varied between −5 and 25 °C. Given this range, the relationship between gaseous N flux and temperature at this site was expressed as an exponential function (Eq. **4**).

[4] $$F_{\mathrm{gaseous N}}{=A\cdot e}^{k\cdot T},$$

where $F_{\mathrm{gaseous}\;N}$ is gaseous N flux (μg N m^−2^ h^−1^), T is the soil temperature (°C), and A and k are fitting parameters. The apparent Q_10_ value of NO emission was calculated using the following equation (Eq. **5**):

[5] $$Q_{10}=e^{10\cdot k}.$$

To determine warming effects on soil profiles, additional soil temperature and moisture probes (not connected to the temperature control system) were installed at 5, 10, 20, 30, and 40 cm depths in the mineral soil in each plot in November 2018 (Campbell CS655 with data logger CR1000, Campbell Scientific, USA). In addition, we set up five temperature and moisture probes in the organic layer (O horizon) of each plot (TOMST, Czech Republic) in April 2023. Precipitation data were obtained from the Qingyuan meteorological station approximately 1 km away from our site.

### Estimating Soil N_2_ Emissions.

Field N_2_ emissions were estimated from a ^15^N-labeling study conducted in 2017, 200 m away from our warming experiment, from which a significant positive linear relationship between soil moisture and the N_2_:N_2_O ratio was observed (*P* = 0.002; *SI Appendix,* Fig. S14). To assess the effects of temperature and moisture on soil N_2_O and N_2_ production under warming, we conducted a complementary incubation experiment in July 2024 using in situ soils from ambient and warming plots. Four grams of sieved soil (2 mm mesh) were amended with ^15^N-labeled NH_4_^15^NO_3_ (50 μg ^15^N g^−1^ soil) in a 20 mL headspace vial, and N_2_O and N_2_ production were measured after a 12-h, dark incubation across a temperature gradient (5, 10, 15, 20, and 25 °C) using gas chromatography (GC-2014, Shimadzu, Japan) and isotope ratio mass spectrometry (Isoprime 100, Isoprime Ltd, UK) (12). The results showed a consistent moisture-driven regulation of the N_2_:N_2_O ratio across treatments, with no significant effect of temperature (*SI Appendix,* Fig. S15).

Based on these findings, we approximated daily N_2_ emissions from the field using this relationship−linking N_2_O:N_2_ ratios to soil moisture in 0 to 10 cm mineral soil—along with measured field N_2_O emissions, according to the following equation (Eq. **6**, derived from the fitting linear regression in *SI Appendix,* Fig S14):

[6] $${\mathrm{Flux}}_{\;N_{2}}\;=\;(0.13\cdot \mathrm{WFPS}\;-\;1.10)\cdot {\mathrm{Flux}}_{\;N_{2}O}.$$

### Soil Extractable C and N and Microbial Biomass C and N.

Soil samples were collected between June and November during growing seasons from 2019 to 2023. Each plot was divided into two subplots, in which four locations were selected randomly for soil sampling. During each sampling event (conducted between 9:00 am and noon), the organic layer (O horizon; 3 to 5 cm depth) was collected using a 10 cm ×10 cm stainless steel frame, followed by mineral soils (0 to 10 cm depth) sampled with a soil corer (5 cm diameter, 10 cm length). Ten grams of sieved soil were extracted with 40 mL of 2 M KCl solution for 1 h (shaking). Extracts were frozen at −20 °C and subsequently analyzed for ammonium (NH_4_^+^-N) and nitrate N (NO_3_^−^-N) using a Smartchem 200 analyzer (Westco Scientific Instruments, Inc., Italy). The contents of soil microbial biomass carbon (MBC) and N (MBN) were determined by chloroform fumigation (57) using a TOC analyzer (TOC, Shimadzu, Kyoto, Japan), with the conversion factors being 0.45 and 0.54 (58, 59), respectively. For extractable organic C (EOC) and extractable organic N (EON), the soil samples were extracted using 0.5 M K_2_SO_4_ (soil: extract 1:4 on a weight basis) and analyzed by a TOC/TN analyzer (Shimadzu, Kyoto, Japan).

### Gene Abundance Associated with Nitrification and Denitrification.

Targeted functional genes were chosen to represent nitrification or denitrification potential involved in processes producing or consuming soil NO and N_2_O, including ammonia-oxidizing archaea (AOA *amoA*), ammonia-oxidizing bacteria (AOB *amoA*), nitrite-reducing denitrifiers (*nirS* and *nirK*), and N_2_O reducing denitrifiers (*nosZ* II). A PowerSoil DNA Isolation Kit (Carlsbad, USA) was used to extract total genomic DNA from 0.25 g of lyophilized soil samples. We measured the concentration and quality of the purified DNA extracts using the Qubit 3.0 fluorometer (Invitrogen) and agarose gel electrophoresis (1.0% agarose gel, 150 V, 40 min). Abundances of AOA *amoA*, AOB *amoA*, *nirS*, *nirK,* and *nosZ* were determined in triplicate using a LightCycler 96 Real-Time PCR System (Roche, Switzerland). The amplification was performed in 20 μL of reaction mixtures, which included 1 μL of DNA template (1 to 10 ng), 10 μL of 2× UltraSYBR mixture (TaKaRa, Japan), 0.5 μL of each primer (10 μM), and 8 μL of sterile distilled water. A list of the primer sequences and quantitative PCR conditions can be found in *SI Appendix*, Table S5. Standard curves were obtained using 10-fold serial dilutions of known copies of plasmids, and negative controls were performed with sterile distilled water. We calculated the abundances of AOA *amoA*, AOB *amoA*, *nirS*, *nirK,* and *nosZ* for each soil sample using individual standard curves. The PCR amplification efficiencies ranged from 92 to 101% for all assays, with R^2^ > 0.99.

### Net N Mineralization and Net Nitrification Rates.

We quantified rates of soil net N mineralization and nitrification for organic horizons and mineral soils (0 to 10 cm depth) during September in the late growing season from 2020 to 2023, noting that gene abundances reflect nitrification/denitrification potentials rather than active enzymatic processes. For each sample, 10 g of freshly sieved soil was weighed into a 50-mL vial. Subsequently, the samples were incubated in a temperature-controlled incubator at 5, 10, 15, 20, and 25 °C for 1 wk. We maintained soil moisture as its initial weight throughout the incubation period. The incubated soil was extracted with 2 M KCl (soil:extract 1:4 by weight) for NH_4_^+^-N and NO_3_^−^-N. Soil net N mineralization and net nitrification rates were calculated according to

[7] $$R_{M}=\left[\left(N_{\mathrm{AA}}\;+\;N_{\mathrm{AN}}\right)\;-\;\left(N_{\mathrm{BA}}\;+\;N_{\mathrm{BN}}\right)\right]/T,$$

[8] $$R_{N}=\;\left(N_{\mathrm{AN}\;}-\;N_{\mathrm{BN}}\right)/T,$$

where N_BA_ and N_BN_ represent the contents of NH_4_^+^-N and NO_3_^−^-N before the incubation, respectively, N_AA_ and N_AN_ represent the contents of NH_4_^+^-N and NO_3_^−^-N after the incubation, R_M_ is the net mineralization rate, R_N_ is the net nitrification rate, and T is the incubation time (7 d).

We estimated the net N mineralization and nitrification rates based on the soil temperatures observed during the plant growing season (May to October) using the temperature sensitivity theory of the Arrhenius equation (Eq. **9**). Growing-season soil temperature in the 0 to 10 cm mineral layer averaged 15 °C under control and 17 °C under warming treatments (Fig. 3*A*).

[9] $$R=a\cdot e^{b\cdot T},$$

where R is the net N mineralization or nitrification rates, T is the incubated temperature (°C), and a and b are fitting parameters.

### N_2_O Emission Response to Warming Forest Soils across Studies.

Data for in situ N_2_O flux measurements from forest soil warming studies were derived from published papers listed in the Web of Science (https://www.webofscience.com), Google Scholar (https://scholar.google.com), and China National Knowledge Infrastructure (https://www.cnki.net). Search terms included nitric oxide, nitrous oxide, dinitrogen, forest soil, and in situ warming. We obtained data from ten warming sites with N_2_O emission measurements in temperate forests (*SI Appendix,* Fig. S1). All the sites only utilized the manual method to observe N_2_O emissions, which offered a lower temporal resolution with the biweekly observation and did not include NO and N_2_ emissions (*SI Appendix,* Table S4). We compiled data on soil N_2_O emissions, mean annual temperatures (MAT), mean annual precipitation (MAP), annual N deposition (N_dep_), soil pH, soil organic C (SOC), soil C/N, soil bulk density (BD), soil temperature (Soil temp), change in soil temperature in response to warming (ΔSoil temp), soil moisture (WFPS), and change in soil moisture induced by warming (ΔWFPS) (*SI Appendix,* Fig. S13). To minimize overrepresentation from multiyear observations at the same site, we calculated the average N_2_O emissions and environmental and substrate variables.

For the response of gaseous N emissions to experimental warming, we used the natural log of the response ratio (lnRR), defined as “effect size” (60). For a given variable with an abnormal distribution, we used log transformations to calculate its warming response ratio according to

[10] $${\mathrm{lnRR}=\log}_{e}\frac{X_{W}}{X_{C}}{=\log}_{e}\left(X_{W}\right)\;{-\;\log}_{e}{(X}_{C}),$$

where the warming response (lnRR) is calculated as the ratio of its averaged value in the warming group (X_W_) to that in the control group (X_C_).

Considering the effect of warming amplitude on the response in different studies, we standardized the response ratio (RRn) to 1 °C warming (61) according to

[11] $$\mathrm{RRn}=\left(\frac{X_{W}\;-\;X_{C}}{X_{C}}\right)/\Delta T,$$

where the standardized warming response (RRn) is calculated as the ratio of its averaged change of variables compared with the control ($\frac{X_{W}\;{-\;X}_{C}}{X_{C}}$) to change in soil temperature in response to warming (ΔT).

### Statistical Analyses.

We used linear regression to assess the relationships between environmental factors (e.g., soil temperature and WFPS) and soil NO and N_2_O fluxes. To reduce variability in the data (62), we averaged soil NO and N_2_O fluxes within temperature intervals of 0.5 °C and WFPS intervals of 1%, prior to relating the fluxes to environmental variables. Data were tested for residual normality using the Shapiro–Wilk test, with a significance level of *P* < 0.05. We used repeated measures ANOVA and mixed-effect models to test the effects of warming and time on soil NO and N_2_O fluxes, soil properties, microbial biomass C and N, and functional genes. Relative effect sizes for soil substrates, functional genes, and microbial biomass were calculated as (warmed–control)/control across all sampling times from 2019 to 2023. Pearson correlation analyses were conducted to determine significant effects of soil properties (soil temperature, moisture, EOC, NH_4_^+^-N, NO_3_^−^-N, and EON), microbial biomass C (MBC) and N (MBN), functional gene abundances, and CO_2_ flux on the averaged gaseous N fluxes over 2 d within each sampling time. All statistical analyses were conducted using R version 4.3.3 (63, 64).

## Supplementary Material

Appendix 01 (PDF)

Movie S1. The video shows the measurement of soil NO and N_2_O fluxes at the Qingyuan forest warming experiment.

## Data Availability

We have deposited the data and source code implementing the analyses on GitHub (https://github.com/KeiFuang/QYWF_Gaseous-N-emission) (65). All data needed to evaluate the conclusions in the paper are present in the paper and/or supporting information.
