# Supplementary material for: Climate warming reduces soil gaseous nitrogen losses in a temperate forest
Source: Proc Natl Acad Sci U S A. 2025 Nov 24;122(48):e2513401122. doi: 10.1073/pnas.2513401122 (PMC12685039; doi:10.1073/pnas.2513401122)
Supplement: Supplementary file 1 — Appendix 01 (PDF) [file pnas.2513401122.sapp.pdf]

Supporting Information for

Climate warming reduces soil gaseous nitrogen losses in a temperate forest

Kai Huang [a, b, c#], Di Wu [a, c#], Dongwei Liu [a, c, d], Yihang Duan [a, c, e], Peter Dörsch [f], Klaus Butterbach-Bahl [g], Xiaoming Fang [a, c], Yuqi Liu [a, h], Chao Wang [a, c], Haoming Yu [i], Lingrui Qu [a, c], Jingwen Xu [a, b, h], Geshere Abdisa Gurmesa [a, c], Ronghua Kang [a, c, d], Shushi Peng [j], Erik A. Hobbie [k], Xiaotang Ju [l], Shuijin Hu [m], Oliver L. Phillips [n], Per Gundersen [o], Weixing Zhu [p], Peter M. Homyak [b*], Yunting Fang [a, c*]

[a] Chinese Academy of Sciences Key Laboratory of Forest Ecology and Silviculture, Institute of Applied Ecology, Chinese Academy of Sciences, Shenyang, Liaoning, China.
[b] Department of Environmental Sciences, University of California, Riverside, CA, 92521, USA.
[c] Key Laboratory of Stable Isotope Techniques and Applications, Shenyang, Liaoning, China.
[d] Qingyuan Forest CERN, National Observation and Research Station, Shenyang, Liaoning, China.
[e] Institute for Global Change Biology and School for Environment and Sustainability, University of Michigan, Ann Arbor, MI 48109, USA.
[f] Faculty of Environmental Sciences and Natural Resource Management, Norwegian University of Life Sciences, Ås, Norway.
[g] Pioneer Center Land--CRAFT, Agroecology, Aarhus University, Aarhus C, Denmark.
[h] University of Chinese Academy of Sciences, Beijing, China.
[i] Laboratory for Earth Surface Processes, College of Urban and Environmental Sciences, Institute of Carbon Neutrality, Peking University, Beijing, China.
[j] Sino-French Institute for Earth System Science, College of Urban and Environmental Sciences, and Laboratory for Earth Surface Processes, Peking University, Beijing, China.
[k] Earth Systems Research Center, Morse Hall, University of New Hampshire, Durham, NH 03824, USA.
[l] School of Tropical Agriculture and Forestry, Hainan University, Haikou, China.
[m] Department of Entomology & Plant Pathology, North Carolina State University, Raleigh, NC, USA.
[n] School of Geography, University of Leeds, Leeds LS2 9JT, UK.
[o] Department of Geosciences and Natural Resource Management, University of Copenhagen, 1958 Frederiksberg C, Denmark.
[p] Department of Biological Sciences, Binghamton University, State University of New York, Binghamton, NY, USA.

#These authors contributed equally: Kai Huang, Di Wu.

*Corresponding authors: phomyak@ucr.edu; fangyt@iae.ac.cn

This PDF file includes:

- Supporting text
- Figures S1 to S15
- Tables S1 to S5
- SI References

Other supporting materials for this manuscript include the following:

- Movies S1

Supporting Information Text

Materials and methods

Auxiliary measurement

In each year, we measured annual tree growth indicators, including diameter at breast height (measured at 1.3 m above the ground) and tree height, in six plots. Total tree woody biomass, encompassing branches, stems, and coarse roots, was calculated using species-specific allometric equations. To quantify litterfall production, two litter traps (1 m × 1 m) were installed per plot to collect litterfall. Annual litterfall production was determined by summing the monthly dry weights of all collected tree species from the traps. Fine root biomass was also estimated as the total weight across all the soil profiles during soil sampling. From July to September each year, we collected fresh tree foliage samples and dried them at 65°C to constant weight. Foliage nitrogen (N) content was measured using an elemental analyzer (Elementar Analysen Systeme GmbH, Germany). Plot-level mean foliage N content was calculated by weighting values according to each species' contribution to total tree biomass contributions.

To quantify soil N leaching, two negative-pressure soil solution collectors (OD21, Prenart Equipment ApS, Denmark) were installed at a depth of 40 cm in each plot to collect soil leachate. Prior to each precipitation event, the collectors were evacuated, and leachate was sampled one week later using light-proof, heat-insulated bottles for analysis. Soil N leaching was quantified using a TOC/TN analyzer (Shimadzu, Kyoto, Japan).

In situ N_2:N_2O ratio measurement

We estimated field N_2 and N_2O emissions using a ^{15}N-labelling experiment across a field soil moisture gradient that consisted of three soil moisture levels, with each level having four replicate plots. A stainless-steel collar (basal area 0.09 m^2; 30 cm × 30 cm) was randomly placed in each plot across the moisture gradient and inserted into the soil down to a 10 cm depth. All collars were installed one week before soil ^{15}N labeling. Stainless-steel chambers (10.5 L; 30 cm × 30 cm × 15 cm), mounted on preinstalled collars, were equipped with a three-way sampling port and a 3-mm diameter pressure equilibration tube (15 cm in length). A solution of labeled $Na^{15}NO_3$ (99.26 atom% ^{15}N diluted in 900 mL deionized (DI) water; Shanghai Research Institute of Chemical Industry, Shanghai, China) was injected into the soil evenly within the collars at a rate of 2.5 g ^{15}N m^{-2} using a syringe with a 10 cm needle. An additional 300 mL of DI water was sprinkled within the collar area to rinse the residual ^{15}N solution from the litter into the soil. After sealing chambers, gas samples were collected at 4-, 7-, 24-, and 30-hours post-labeling using 100 mL gas sampling bags.

The concentration and the isotopic signature of N_2O and N_2 were measured using a gas chromatography (GC-2014, Shimadzu, Japan) and an isotope ratio mass spectrometer (IRMS, IsoPrime 100, UK) coupled with an auto-sampler of 112 slots (Gilson GX-271, UK). Based on these measurements, we calculated the soil N_2:N_2O emission ratios and their relationship with soil moisture.

Data analyses

We used repeated measures analysis of variance (ANOVA) to test the effects of warming and time on soil N leaching, plant foliage N concentration, plant woody biomass, root biomass, and litterfall production. All statistical analyses were performed using the statistical software R version 4.3.3.

Figures

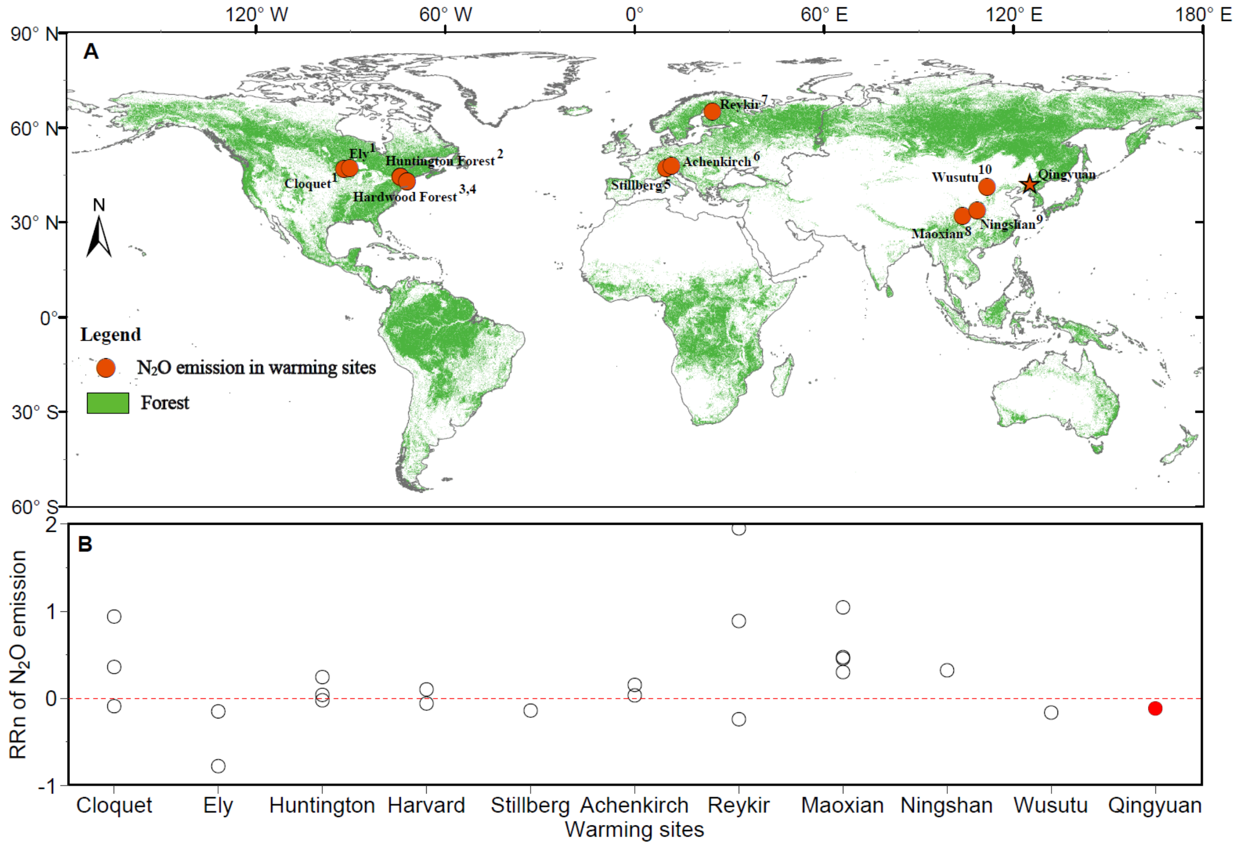

Fig. S1. Global patterns and N_2O emission responses (RRn) in forest warming experiments. The red dots represent the locations of reported N_2O emissions from forest warming experiments, with the number in the upper right corner of each site indicating the reference number (panel **A**). The standard warming response of N_2O emissions (RRn) is presented in panel **B**. Different dots at the same site represent varying warming intensities and combinations of warming and precipitation manipulation treatments. Our in situ soil warming study was conducted in the Qingyuan temperate forest, China.

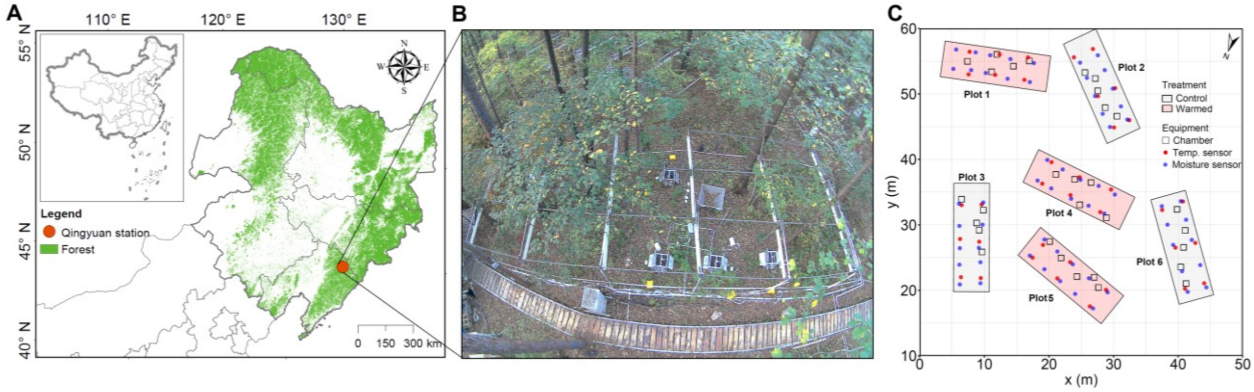

Fig. S2. Experimental set-up of soil warming in the Qingyuan temperate forest. The green areas on the map denote the extent of forest coverage, with the red dot indicating the location of our study site in the Qingyuan Forest Station, northeast China **(A)**. Photograph of the infrared warming experiment **(B)**. Layout of the six experimental plots, each assigned to one of two climate treatments: warmed and control, with five automated gas flux chambers, six soil temperature sensors, and ten soil moisture sensors installed in each plot **(C)**.

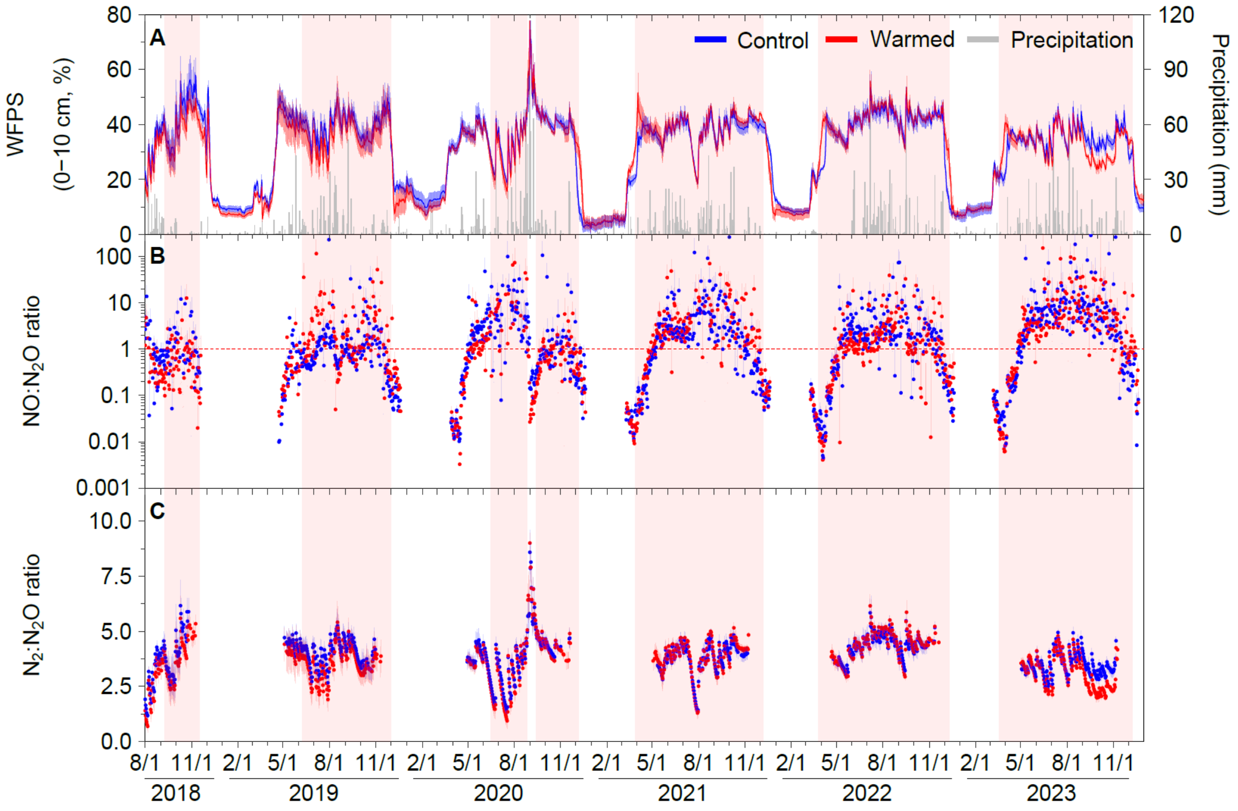

Fig. S3. Daily precipitation and soil water-filled pore space (WFPS) in the 0-10 cm mineral layer, the ratio of NO to N_2O fluxes, and the N_2:N_2O ratio. The red and blue solid lines represent the warmed and control in all the panels. The grey bar denotes the daily precipitation in panel **A**. The light red background represents the warming period in all the panels **(A-C)**.

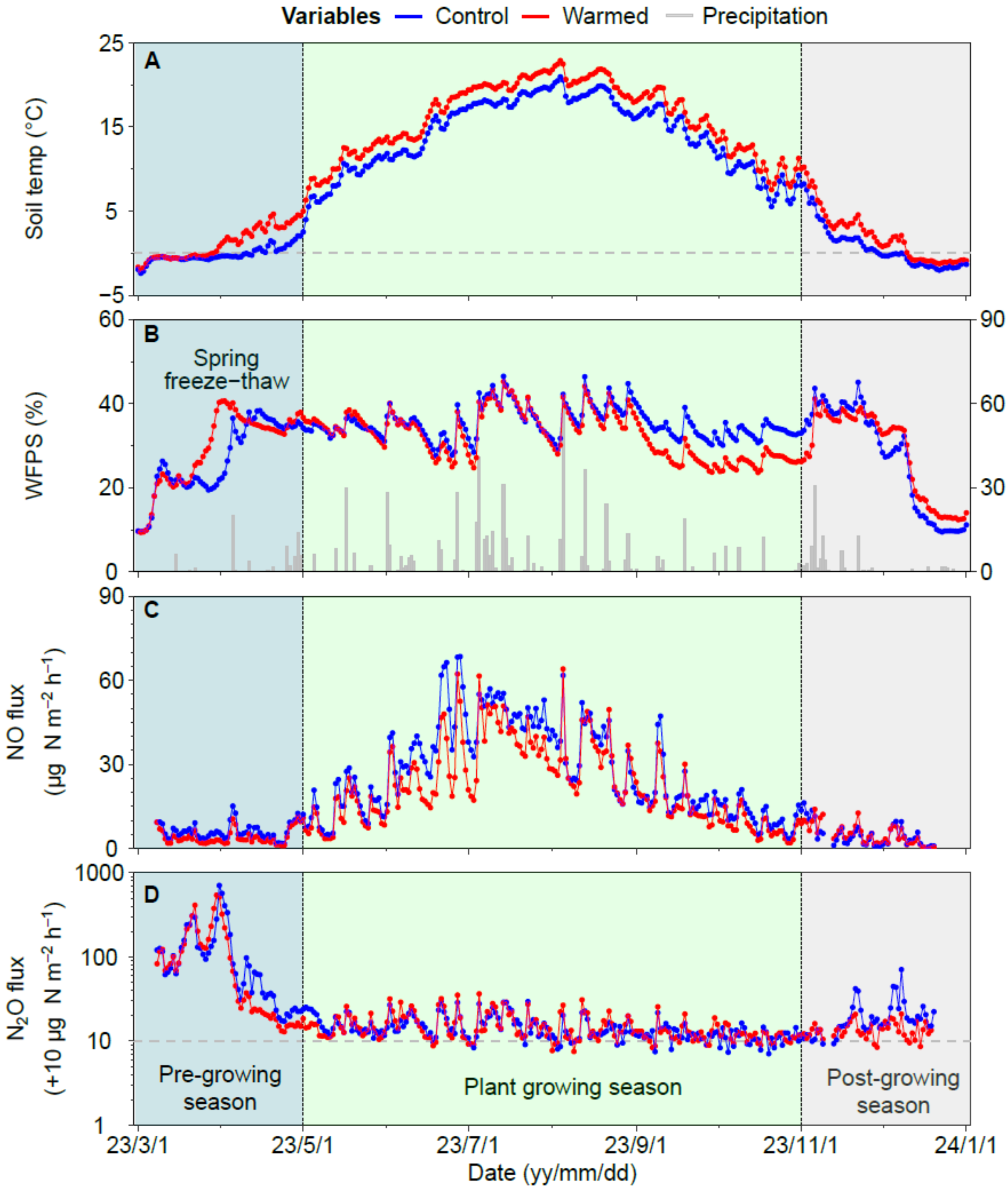

Fig. S4. Seasonal variations in soil temperature, WFPS, NO and N_2O fluxes in 2023. The periods were divided into the plant pre-growing season (March to April), the plant growing season (May to October) and the plant post-growing season (November to December). The blue line represents control plots, and the red line represents warmed plots. The grey bar denotes the daily precipitation in panel **(B)**.

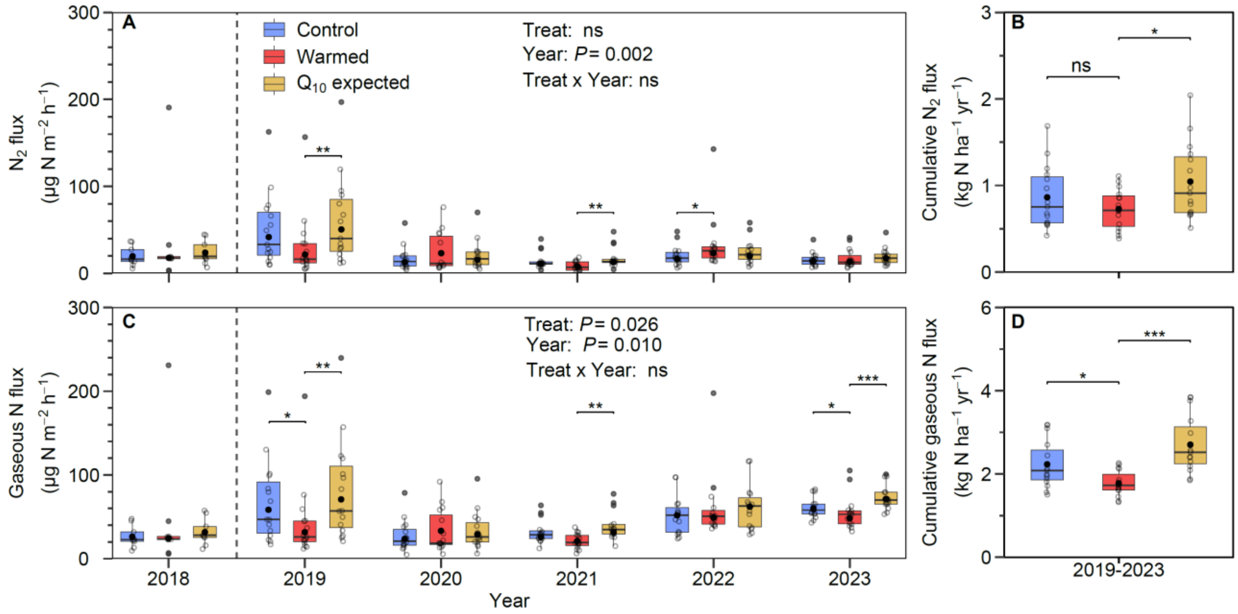

Fig. S5. The response of N_2 and gaseous N ($NO+N_2O+N_2$) emissions to warming during the study period. The fluxes in 2018 represent the pre-treatment period, with the fluxes from 2019 to 2023 representing the five years of warming **(A, C)**. Cumulative gaseous N fluxes from 2019 to 2023 **(B, D)**. Colors represent different treatments (blue = control, red = warming, and orange = Q_{10}-expected fluxes). Q_{10}-expected fluxes for N_2 were calculated based on their Q_{10} values. Grey dots represent the average values for each chamber, while black dots represent the average values for each group (n = 15 chambers). Repeated measures ANOVA was used to test the effects of warming treatment and time on soil NO and N_2O fluxes. Statistical significance between control and warming treatment is denoted by asterisk (*P < 0.05, **P < 0.01, ***P < 0.001) or ns (non-significant).

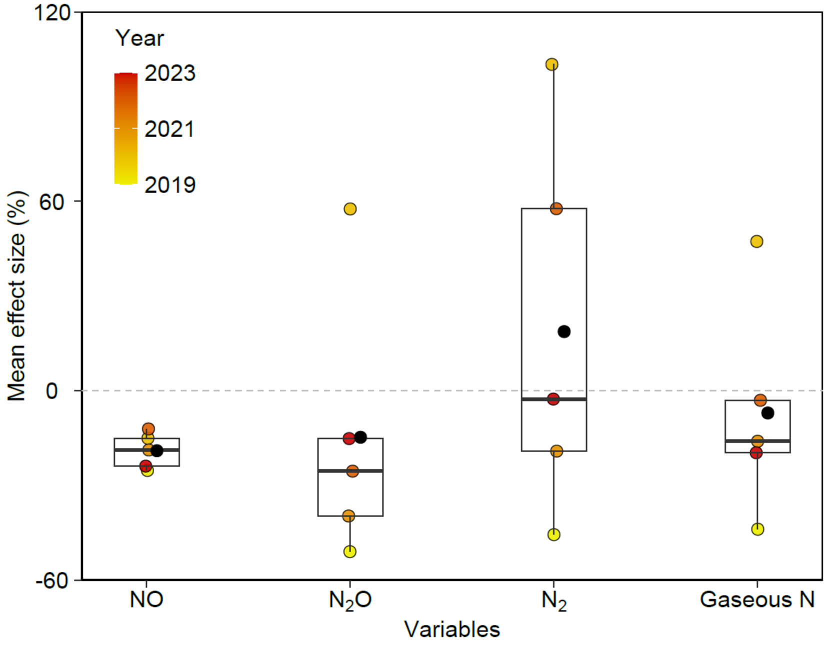

Fig. S6. Warming effects on the normalized changes in gaseous N emissions from 2019 to 2023. Effect sizes were calculated by (warmed−control)/control. Jittered dots, colored from yellow to red, represent the means for each year, while black dots indicate the overall mean changes across five years. Box plots are standard Tukey plots, where the center line represents the median, the lower and upper lines represent the first and third quartiles, and whiskers represent +1.5 times the interquartile range.

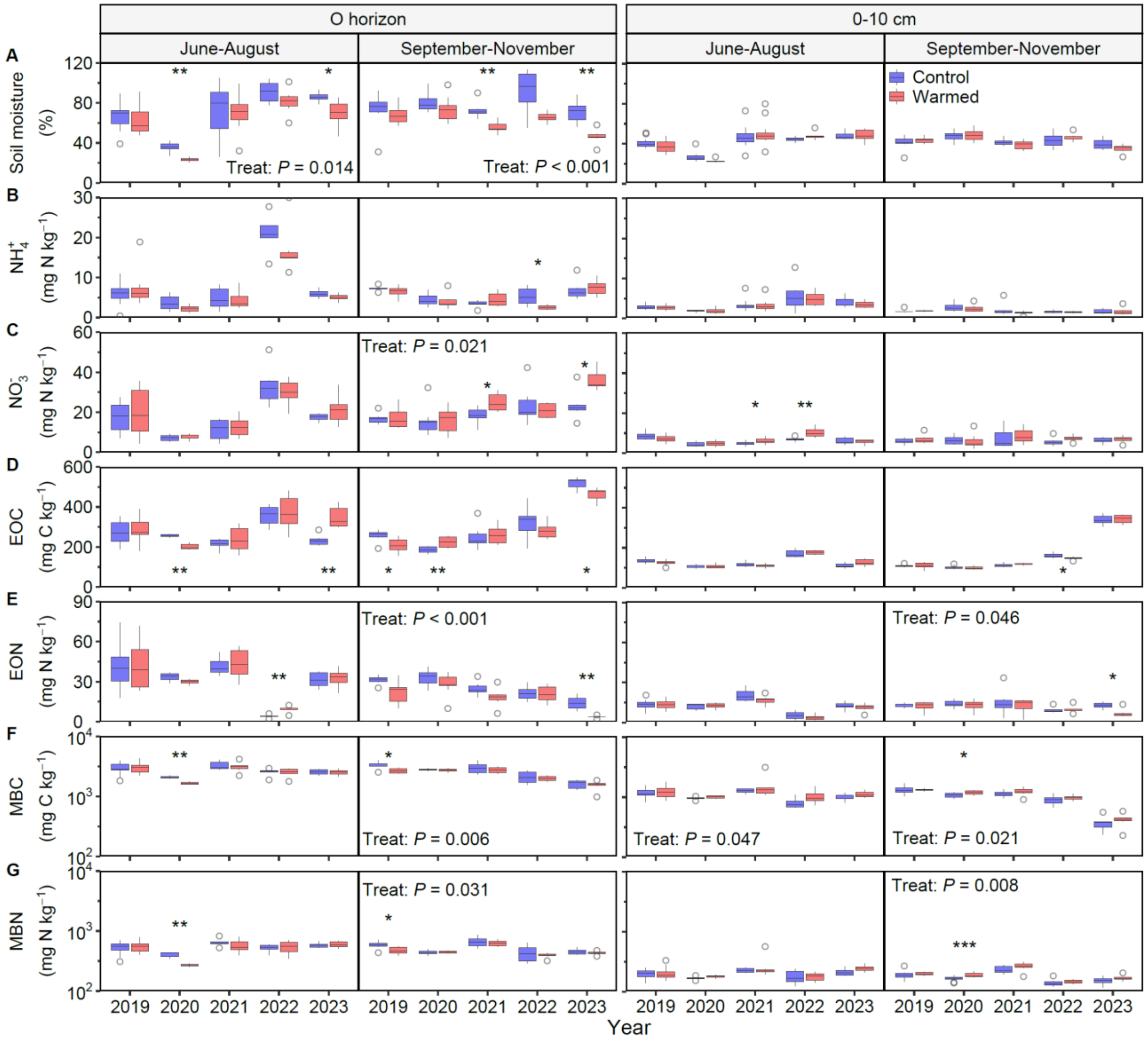

Fig. S7. Seasonal variations in soil moisture (gravimetric water content), soil extractable ammonium and nitrate concentrations, extractable organic C (EOC), soil extractable organic N (EON), microbial biomass C (MBC), and microbial biomass N (MBN) in the organic horizon and the 0-10 cm mineral soil in response to warming. Repeated measures ANOVA was used to test the effects of warming treatment on soil parameters. The level of significance is determined by two-sided t-test: **P* < 0.05, ***P* < 0.01 and ****P* < 0.001.

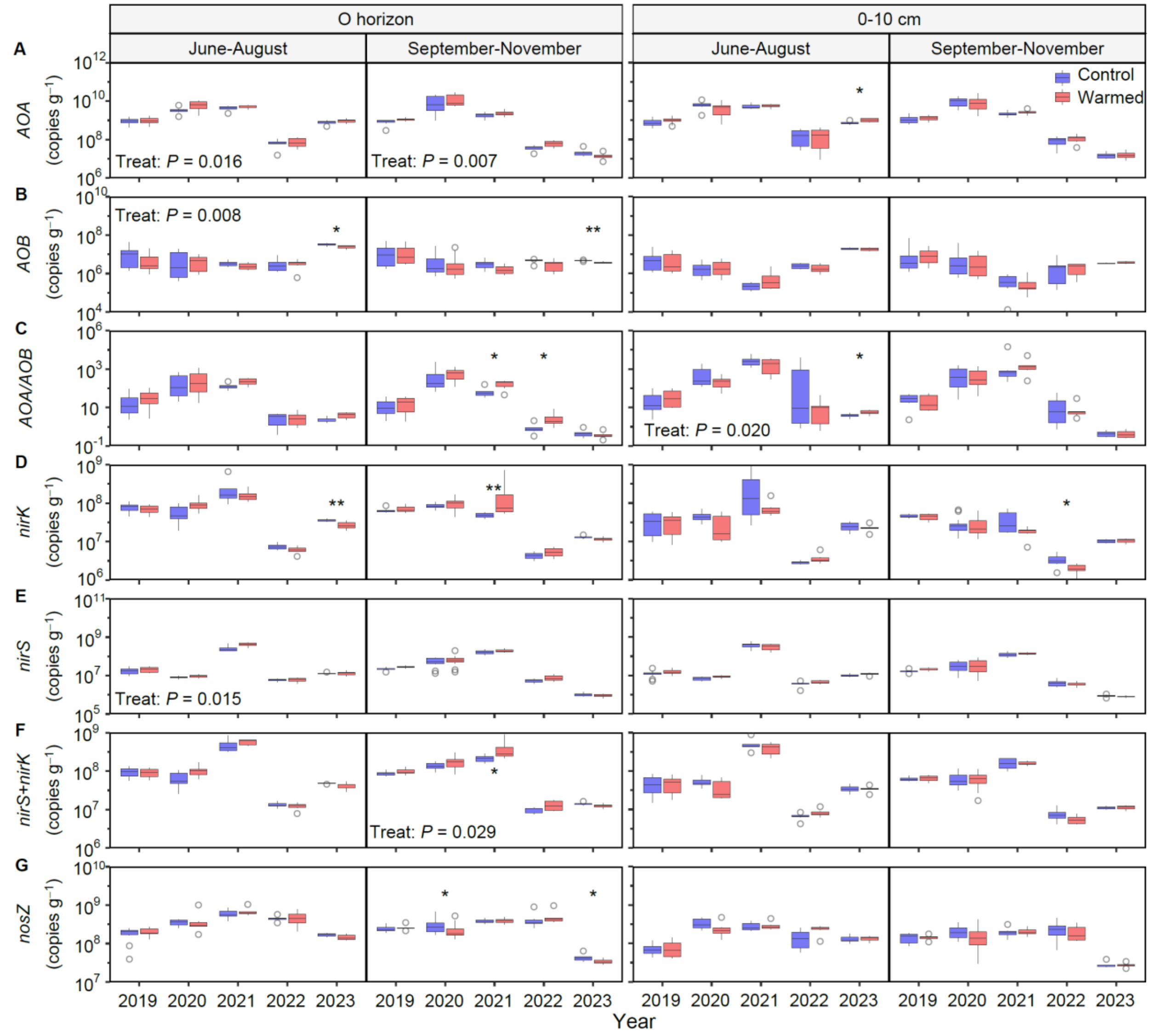

Fig. S8. Warming effects on soil functional genes involved in N transformations in the organic horizon and 0-10 cm mineral layer. Repeated measures ANOVA was used to test the effects of warming treatment and time on soil functional genes associated with microbial nitrification and denitrification processes. The level of significance is determined by two-sided t-test: *$P < 0.05$, **$P < 0.01$ and ***$P < 0.001$.

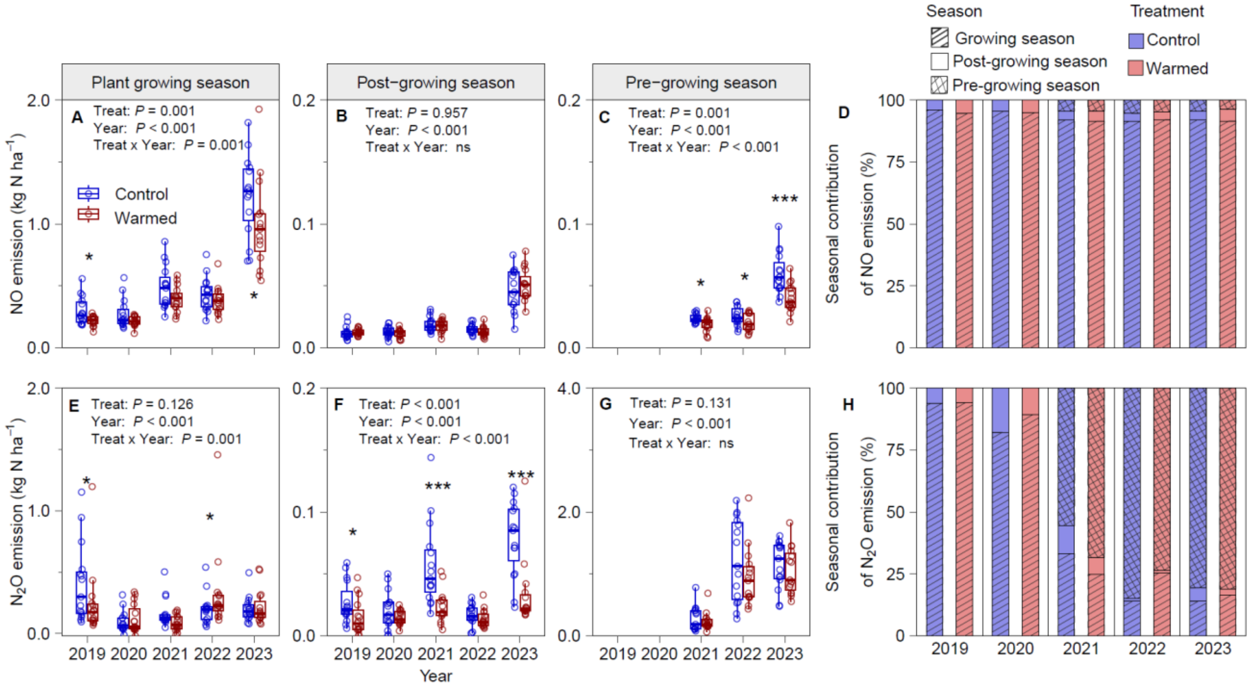

Fig. S9. Seasonal responses of soil NO and N_2O emissions to warming. The seasons were divided into the plant growing season (May to October), the plant post-growing season (November to December) and the plant pre-growing season (March to April) from 2019 to 2023. All data analyses were based on measurements taken during the warming period, where repeated measures ANOVA tested the effects of warming treatment and time on seasonal NO and N_2O emissions **(A–C, E–G)**. The bar with slashes denotes the contribution of the plant growing season **(D, H)**. The level of significance is determined by two-sided t-test: *$P < 0.05$, **$P < 0.01$ and ***$P < 0.001$.

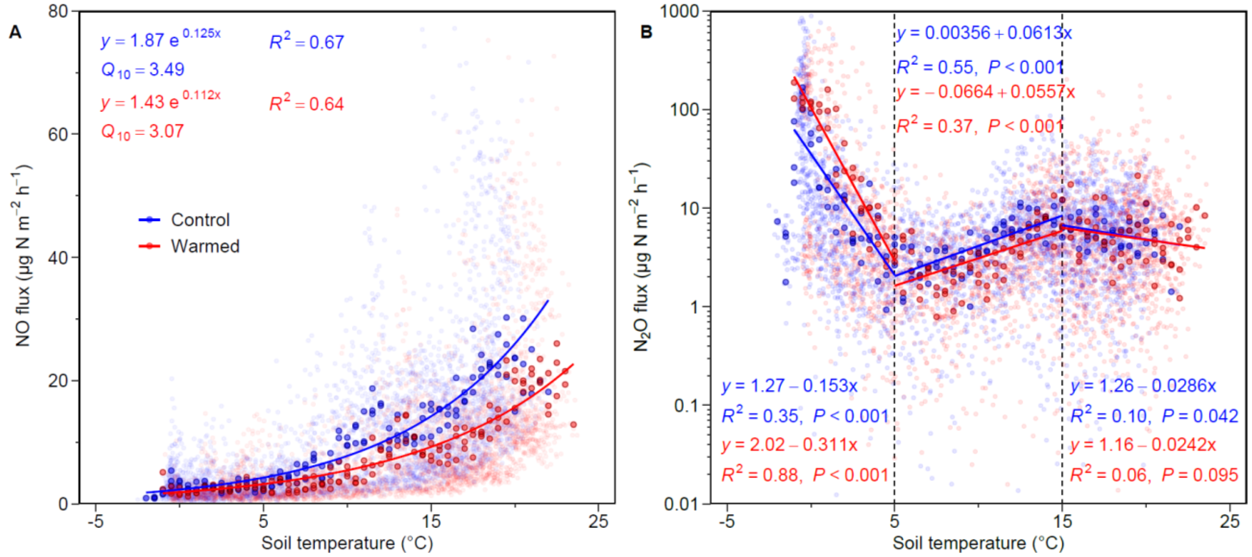

Fig. S10. Relationships of NO and N_2O fluxes with soil temperature (5 cm mineral depth) during the entire warming period. The red and blue lines represent the fitting relationships between NO or N_2O fluxes and soil temperature in warmed and control plots, using recalculated data (bold dots) that were binned at 0.5°C intervals.

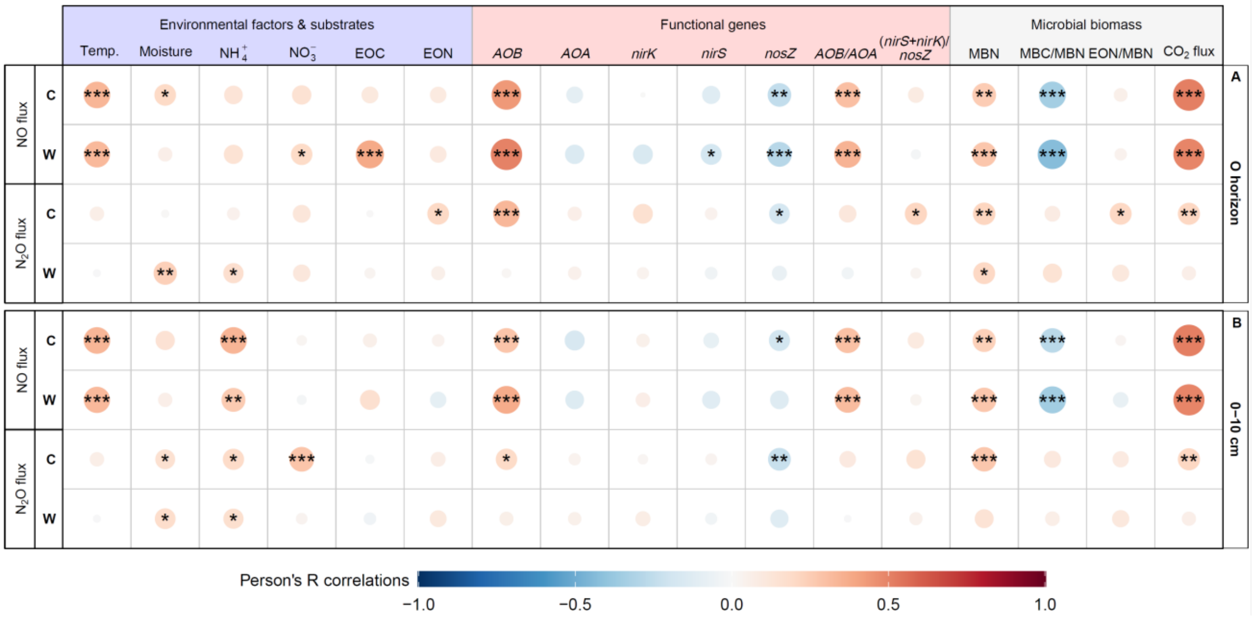

Fig. S11. Pearson correlation analysis of NO and N_2O fluxes with soil characteristics, including soil properties, microbial biomass N (MBN), functional gene abundances, and soil CO_2 fluxes. These analyses were performed separately for control (C) and warmed (W) treatments across all sampling timepoints. The correlation coefficients, from negative to positive, are indicated by color intensity ranging from red to blue**.** The level of significance is determined by two-sided t-test: *$P < 0.05$, **$P < 0.01$ and ***$P < 0.001$.

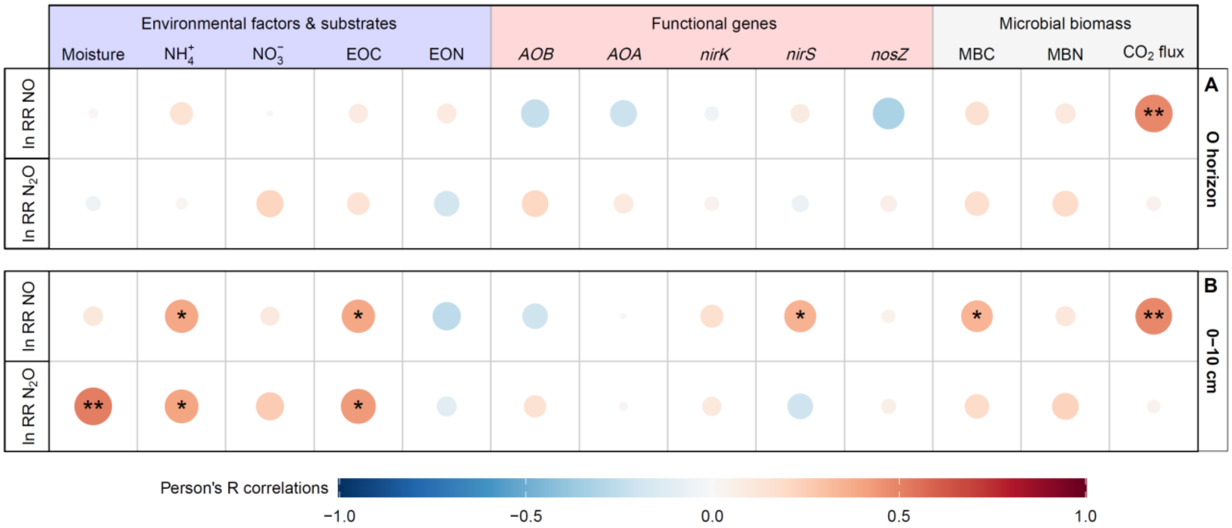

Fig. S12. Pearson correlations between warming-induced changes in soil NO and N_2O emissions and responses of soil moisture, substrates, and microbial functional genes during the 2019-2023 warming experiment. These analyses were performed across all sampling timepoints. The correlation coefficients, from negative to positive, are indicated by color intensity ranging from red to blue. The level of significance is determined by two-sided t-test: **P* < 0.05, ***P* < 0.01 and ****P* < 0.001.

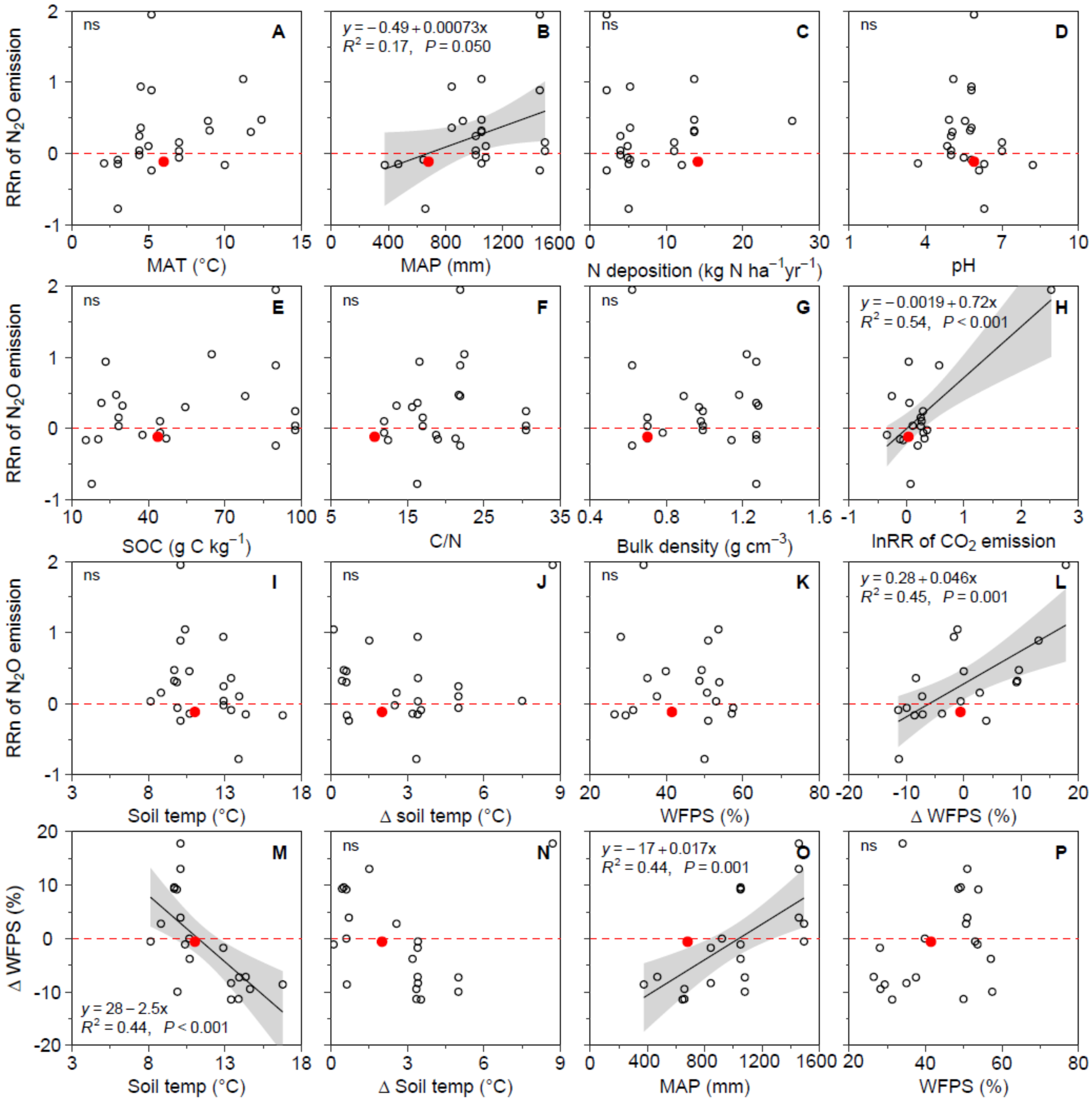

Fig. S13. Correlations of the standard warming response of N_2O emissions (RRn) with climate and soil factors across forest ecosystems under experimental warming. Data are collected from eleven warming sites, encompassing various warming intensities or combinations of warming and precipitation manipulation experiments (n = 24). The red dot represents this study in the Qingyuan forest warming experiment. Shaded regions indicate the 95% confidence interval for the linear regression for RRn **(A-L)** and ΔWFPS **(M-P)** with variables.

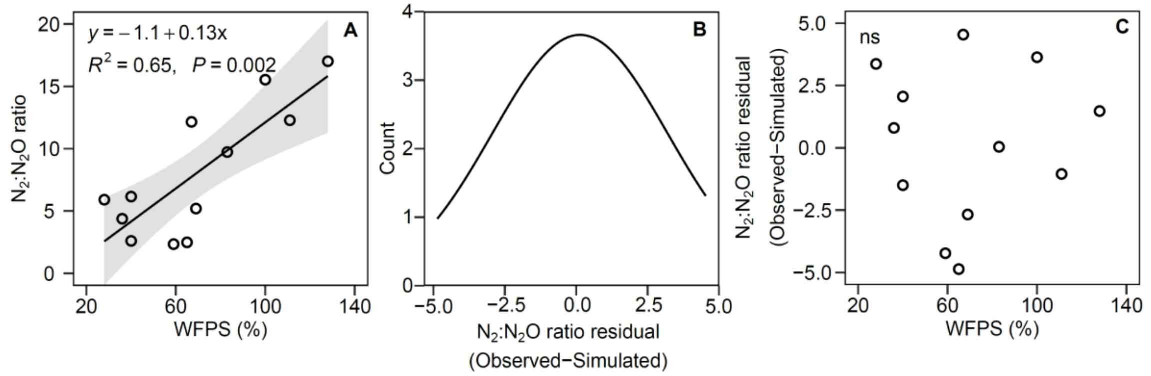

Fig. S14. The ratio of N_2:N_2O as a function of soil water-filled pore space (WFPS). Data were derived from a field experiment using ^{15}N-NO_3^- labelling in Qingyuan mixed forest, approximately 200 m from our warming plots.

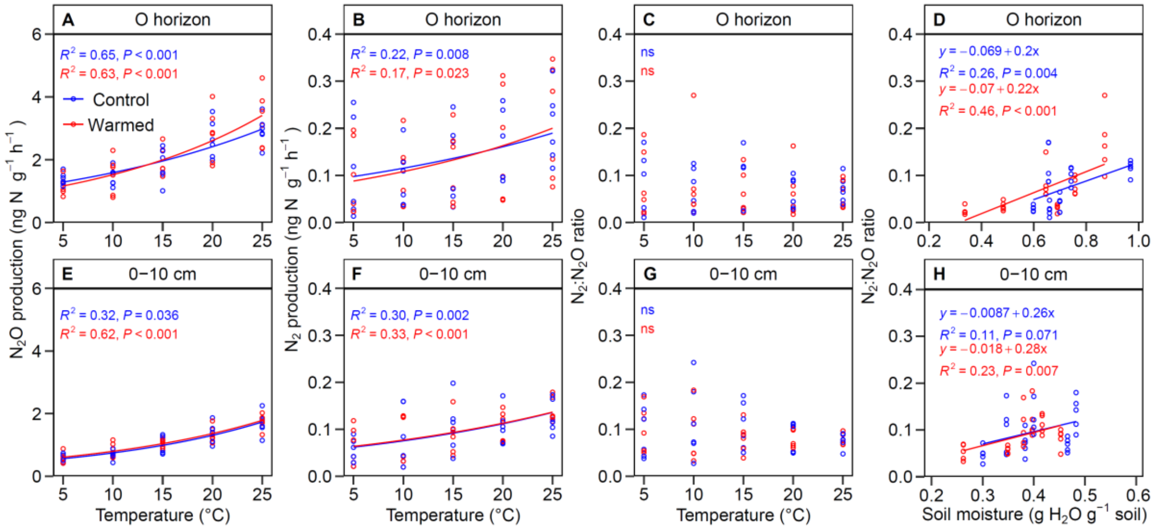

Fig. S15. Warming effects on soil N_2O and N_2 production and the N_2:N_2O ratio. Gaseous N emissions were measured after a 12-hour laboratory incubation under a controlled temperature gradient (5 to 25°C), using soil samples from our experimental warming plots. An exponential model was used to analyze the relationship between gaseous N production and temperature **(A-B, E-F)**.

Table S1. Response of soil net N mineralization and nitrification rates to warming during the plant growing season (05/01-10/31) from 2020 to 2023. Shown are mean values with standard errors in parenthesis (n = 6 subplots).

Years	Soil layers	Net N mineralization rate (kg N ha^{-1})		Net nitrification rate (kg N ha^{-1})	
		control	warmed	control	warmed
2020	O horizon	17 (4)	13 (1)	22 (5)	20 (3)
	0-10 cm	131 (26)	102 (13)	166 (26)	130 (15)
	full profile	148	115	188	150
2021	O horizon	44 (7)	38 (6)	42 (5)	42 (3)
	0-10 cm	215 (51)	189 (6)	241 (67)	198 (5)
	full profile	259	227	283	240
2022	O horizon	24 (2)	16 (2)	38 (3)	27 (3)
	0-10 cm	88 (7)	95 (9)	113 (6)	126 (8)
	full profile	112	112	151	153
2023	O horizon	12 (3)	10 (2)	14 (2)	12 (2)
	0-10 cm	106 (12)	97 (7)	99 (10)	89 (8)
	full profile	118	107	113	101

Table S2. Cumulative soil NO and N_2O emissions in Qingyuan temperate forest. Emissions are shown as the average with standard errors in parenthesis (n = 15 chambers). The plant growing season extended from 05/01-10/31 and the plant dormant season from 11/01-04/30.

Gaseous N	Periods	Emissions (kg N ha^{-1} yr^{-1})				
		2019	2020	2021	2022	2023
NO	growing season	0.33 (0.03)	0.40 (0.03)	0.48 (0.04)	0.45 (0.04)	1.30 (0.09)
	dormant season	0.02 (0.00)	0.03 (0.00)	0.06 (0.00)	0.04 (0.00)	0.06 (0.01)
	all	0.35	0.43	0.54	0.50	1.36
N_2O	growing season	0.47 (0.09)	0.25 (0.04)	0.16 (0.03)	0.20 (0.03)	0.20 (0.03)
	dormant season	0.12 (0.03)	0.53 (0.08)	0.94 (0.11)	1.53 (0.19)	1.53 (0.12)
	all	0.59	0.78	1.10	1.73	1.73

Table S3. The response of plant biomass and soil N leaching to warming from 2019 to 2023. All the values are presented as the average with standard errors in parenthesis (n = 3 plots).

Year	Treatment	Foliage N content ($g\ N\ kg^{-1}$)	Litterfall production ($10^3\ kg\ ha^{-1}$)	Total woody biomass ($Mg\ ha^{-1}$)	Fine root biomass ($10^3\ kg\ ha^{-1}$)	Soil N leaching ($mg\ N\ L^{-1}$)
2018	control	nd	5.6 (0.5)	296 (63)	nd	nd
	warmed	nd	6.1 (0.4)	271 (49)	nd	nd
2019	control	22.7 (0.6)	6.0 (0.5)	306 (64)	5.8 (0.9)	3.8 (1.2)
	warmed	23.4 (0.4)	6.0 (0.4)	282 (49)	6.1 (0.8)	3.1 (1.6)
2020	control	18.4 (1.0)	6.4 (0.9)	314 (66)	8.4 (0.9)	3.5 (0.5)
	warmed	18.8 (1.0)	6.6 (0.9)	290 (51)	9.3 (2.3)	3.5 (0.6)
2021	control	26.3 (1.7)	5.7 (0.2)	323 (68)	3.2 (1.1)	nd
	warmed	25.8 (1.1)	6.1 (0.3)	299 (52)	3.6 (1.1)	nd
2022	control	25.2 (0.9)	5.8 (0.1)	331 (70)	1.8 (0.7)	nd
	warmed	25.7 (2.1)	7.3 (1.0)	308 (53)	1.5 (0.5)	nd
2023	control	nd	6.5 (0.1)	340 (73)	2.3 (0.3)	nd
	warmed	nd	6.4 (0.1)	317 (54)	2.5 (0.4)	nd

Table S4. Summary of the studies on gaseous nitrogen emissions to in situ warming.

Reference	Country/Site	Location	Forest type	Duration	Warming method	Precipitation ($mm\ yr^{-1}$)	Monitoring method	N trans-formation	Gene abundance	Gaseous N emissions to warming (measurements)		
										N_2O	NO	N_2
Martins et al., 2017	US/ Cloquet	46.67°N 92.52°W	Temperate mixed forest, 50 yr	2013	Infrared heater+ Heating cable, +3.4°C	842	Manual, monthly		✓	✓ 36		
Martins et al., 2017	US/ Ely	46.93°N 91.75°W	Temperate mixed forest, 50 yr	2013	Infrared heater+ Heating cable, +3.4°C	658	Manual, monthly		✓	✓ 36		
McHale et al., 1998	US/ Huntington Forest	43.98°N 74.23°W	Temperate mixed forest	1993-1994	Heating cable, +2.5°C; +5°C; +7.5°C	1010	Manual, biweekly			✓ 80		
Peterjohn et al., 1994; Butler et al., 2012	US/ Hardwood Forest	42.50°N 72.17°W	Temperate mixed forest	1994, 2003-2007	Heating cable, +5°C	1080	Manual, monthly	✓		✓ 72		
Karbin et al., 2015	Switzerland/ Stillberg	46.77°N 9.87°E	Temperate mixed forest, 40 yr	2011-2012	Heating cable, +3.2°C	1050	Manual, monthly			✓ 40		
Heinzle et al., 2023	Austria/ Achenkirch	47.57°N 11.63°E	Temperate mixed forest, 130 yr	2006-2009, 2019-2021	Heating cable, +3.0°C	1493	Manual, monthly			✓ 606		*
Maljanen et al., 2018	Iceland/ Reykir	64.78°N 24.63°E	Northern spruce, 50 yr	2016	Geothermal, +0.7°C; +1.5°C; +8.7°C	1457	Manual, once			✓ 12		
Li et al., 2019	China/ Maoxian	31.68°N 103.88°E	Alpine coniferous forest	2013-2014	Infrared heater, +3.7°C	920	Manual, monthly			✓ 128		
Wang et al., 2021	China/ Ningshan	33.38°N 108.50°E	Temperate mixed forest, 50 yr	2016-2018	OTCs, +0.2°C	1050	Manual, biweekly			✓ 630		
Liang et al, 2022	China/ Wusutu	40.85°N 111.57°E	Temperate pine forest, 30 yr	2020	OTCs, +0.6°C	375	Manual, biweekly			✓ 216		
This study	China/ Qingyuan	41.85°N 124.90°E	Temperate mixed forest, 70 yr	2018-Now	Infrared heater, +2°C	680	Automatic, hourly	✓	✓	✓ 200,000	✓ 200,000	*

Note: Soil nitrogen (N) transformation encompasses soil N mineralization and nitrification rates, while functional genes refer to the key genes associated with nitrification and denitrification processes. The symbol "✓" indicates measured response in each study, with the numbering below indicates the amount of measurement. "*" denotes estimated responses.

Table S5. Real-time PCR primer sets and thermocycling conditions used in this study.

Target gene	Primer	Nucleotide sequence (5'-3')	qPCR program	Reference
amoA-Archaea	amoAF	STAATGGTCTGGCTTAGACG	1 cycle: 95˚C 30s; 45 cycles: 95˚C 10 s, 60˚C 30 s, 72˚C 45s;	(11)
	amoAR	GCGGCCATCCATCTGTATGT	melting curve	
amoA-Bacteria	amoA-1F	GGGGTTTCTACTGGTGGT	1 cycle: 95˚C 30s; 45 cycles: 95˚C 10 s, 60˚C 30 s, 72˚C 45s;	(12)
	amoA-2R	CCCCTCKGSAAAGCCTTCTTC	melting curve	
nirK	nirK 1F	GGMATGGTKCCSTGGCA	1 cycle: 95˚C 30s; 45 cycles: 95˚C 10 s, 55˚C 30 s, 72˚C 40s;	(13)
	nirK 5R	GCCTCGATCAGRTTRTGGTT	melting curve	
nirS	nirS cd3AF	GTSAACGTSAAGGARACSGG	1 cycle: 95˚C 30s; 45 cycles: 95˚C 10 s, 57˚C 30 s, 72˚C 40s;	(14)
	nirSR3cd	GASTTCGGRTGSGTCTTGA	melting curve	
nosZ	nosZ 2F	CGCRACGGCAASAAGGTSMSSGT	1 cycle: 95˚C 30s; 45 cycles: 95˚C 10 s, 60˚C 30s; melting curve	(15)
	nosZ 2R	CAKRTGCAKSGCRTGGCAGAA		

Movie S1 (separate file). The video shows the measurement of soil NO and N_2O fluxes at the Qingyuan forest warming experiment.

SI References

1. Martins C. S. C. et al., Identifying environmental drivers of greenhouse gas emissions under warming and reduced rainfall in boreal-temperate forests. Functional Ecology 31:2356-2368 (2017).
2. McHale P. J. et al., Soil warming in a northern hardwood forest: trace gas fluxes and leaf litter decomposition. Canadian Journal of Forest Research 28, 1365-1372 (1998).
3. Peterjohn W. T. et al., Responses of trace gas fluxes and N availability to experimentally elevated soil temperatures. Ecological Applications 4:617-625 (1994).
4. Butler S. M. et al., Soil warming alters nitrogen cycling in a New England forest: implications for ecosystem function and structure. Oecologia 168, 819-828 (2012).
5. Karbin S. et al., Treeline soil warming does not affect soil methane fluxes and the spatial micro-distribution of methanotrophic bacteria. Soil Biology Biochemistry 86, 164-171 (2015).
6. Heinzle J. et al., Soil CH_4 and N_2O response diminishes during decadal soil warming in a temperate mountain forest. Agricultural and Forest Meteorology 329 (2023).
7. Maljanen M. et al., The emissions of nitrous oxide and methane from natural soil temperature gradients in a volcanic area in southwest Iceland. Soil Biology Biochemistry 109, 70-80 (2017).
8. Li D. et al., Differential Responses and Controls of Soil CO_2 and N_2O Fluxes to Experimental Warming and Nitrogen Fertilization in a Subalpine Coniferous Spruce (Picea asperata Mast.) Plantation Forest. Forests 10, (2019).
9. Wang X. et al., Effects of warming on soil N_2O Flux in Qingling temperate forest. Journal of Southwest Forestry University, 41(4): 35-41 (2021).
10. Liang L. et al., Effects of litter on soil greenhouse gas flux of pinus tabulaeformis plantation in Daqing Mountain under simulated warming (in Chinese). Ecology and Environmental Sciences 31, 478-486 (2022).
11. Braker G. et al., Development of PCR primer systems for amplification of nitrite reductase genes (nirK and nirS) to detect denitrifying bacteria in environmental samples. Applied and Environmental Microbiology 64, 3769-3775 (1998).
12. Francis C. A. et al., Ubiquity and diversity of ammonia-oxidizing archaea in water columns and sediments of the ocean. Proceedings of the National Academy of Sciences of the United States of America 102, 14683-14688 (2005).
13. Henry S. et al., Quantitative detection of the nosZ gene, encoding nitrous oxide reductase, and comparison of the abundances of 16S rRNA, narG, nirK, and nosZ genes in soils. Applied and Environmental Microbiology 72, 5181-5189 (2006).
14. Muyzer G. et al., Profiling of complex microbial populations by denaturing gradient gel electrophoresis analysis of polymerase chain reaction-amplified genes coding for 16S rRNA. Applied and Environmental Microbiology 59, 695-700 (1993).
15. Rotthauwe J. H. et al., The ammonia monooxygenase structural gene amoA as a functional marker: Molecular fine-scale analysis of natural ammonia-oxidizing populations. Applied and Environmental Microbiology 63, 4704-4712 (1997).
